# Differences in visual search behavior between expert and novice individual sports athletes: a systematic review with meta-analysis

**DOI:** 10.3389/fphys.2026.1793747

**Published:** 2026-05-14

**Authors:** Qiangquan Wang, Wei Guo, Dongxu Gao, Youlin Xiao, Yukun Song, Xiushen Dong, Guowei Yuan, Tao Jiang

**Affiliations:** 1 Ningxia Normal University, Guyuan, China; 2 Tsinghua University, Beijing, China; 3 Dalian University, Dalian, China; 4 Loughborough University, Loughborough, United Kingdom; 5 Hunan Normal University, Changsha, China; 6 Xinjiang Medical University, Urumqi, China

**Keywords:** attention, decision-making, expertise, eye movements, gaze behavior, motor behavior

## Abstract

**Abstract:**

This systematic review and meta-analysis examined expert–novice differences in visual search behaviour in individual-sport athletes, addressing a gap in a literature that has focused primarily on team sports. Following PRISMA 2020 guidelines, we searched EBSCO, PubMed, Scopus, and SPORTDiscus databases up to November 1, 2025, identifying 26 eligible studies (total N = 611 athletes). Key outcomes included chronological age, years of practice, number of fixations, fixation duration, number of fixation locations, fixation positions, and quiet eye duration. Meta-analyses were conducted using standardized mean differences (SMD) with random-effects models in Review Manager 5.4. Experts had significantly more years of practice than novices (SMD = 3.45, 95% CI 1.82–5.08, p < 0.0001; very large effect).Compared with novices, experts showed fewer fixations (SMD = −1.52, 95% CI −2.38 to −0.65, p = 0.0006; very large effect), longer fixation durations (SMD = 0.93, 95% CI 0.44–1.41, p = 0.0002; large effect), and fewer distinct fixation locations (SMD = −1.27, 95% CI −2.26 to −0.28, p = 0.01; very large effect).Subgroup analyses by sport type showed no significant moderating effects. Visual search strategies are highly task-specific. Combat experts prioritize central body regions to optimize peripheral vision, whereas badminton and tennis experts focus on distal cues (e.g., arm and racket) and kinematic information (e.g., trunk and hips), respectively. These variations reflect selective processing of key perceptual cues under unique sporting constraints. Risk of bias, assessed using RoBANS, indicated moderate concerns in confounding variables. Overall, these findings suggest that experts employ more efficient visual search strategies, supporting the development of perceptual–cognitive training interventions in individual sports.

**Systematic Review Registration:**

https://www.crd.york.ac.uk/PROSPERO/view/CRD420261280358, identifier CRD420261280358.

## 1 Introduction

Decision-making is defined as an individual’s ability to select the most appropriate action from multiple possibilities to achieve a specific goal (Hastie, 2001). In competitive sports, decision-making ability has been shown to be a key factor in athletes achieving optimal performance (Gréhaigne et al., 2001; Forsman et al., 2016). During competition, athletes are required to continuously interpret environmental information and adjust their behavior and strategies in real time in response to opponents’ actions and contextual factors (Araujo and Davids, 2016).

Higher-quality decision-making relies on a series of ongoing perceptual-cognitive skills, including identifying and extracting key environmental informantion, integrating it with existing knowledge structures, and selecting and executing appropriate responses (Marteniuk, 1976). In sports contexts, these abilities largely depend on athletes’ visual search strategies, specifically, deciding “when to look and where to look” in complex and dynamic scenarios. Evidence suggests that visual search behaviour is a critical factor distinguishing expert from novice peformers (Le Runigo et al., 2005). Because visual environments are often information-rich and contain numerous task-relevant or irrelevant cues, the ability to effectively filter and use key information is essential for sports performance (Mann et al., 2007).

Studies have shown that, under certain conditions, gaze shifts are often accompanied by reallocations of attention, with a close association between the two (Shepherd et al., 1986; Kowler et al., 1995; Henderson, 2003). However, for a long time, it was believed that shifts in attention could occur independently of gaze shifts, leaving the relationship between them unclear (Posner, 1980). With the advances in eye-tracking research methodology, accumulating evidence suggests that when individuals saccade their gaze to a new spatial location, attention also shifts synchronously toward the saccade target location. In other words, when athletes redirect their gaze to a new area, their attention is at least briefly focused on that area (Vickers, 2009). This gaze-attention coupling may also be influenced by the structure of the visual field. Existing research had tended to emphasise foveal vision while comparatively neglecting the comtribution of peripheral vision. However, central vision covers only about 5° of the visual field, despite having the highest visual acuity, which supports fine discrimination of key visual stimuli (Millodot, 2017). By contrast, peripheral vision, although lower in resolution, plays a crucial role in visual processing, particularly in integrating contextual information and perceiving spatial structures (Rosenholtz, 2016).

Eye movement analysis in sports contexts typically foucuses on three core variables: fixations, saccades, and smooth pursuits (Bojko, 2013). When examining fixation behaviour, researchers commonly quantify location (the specific cues the individual is attending to), duration (the time spent on that cue), and onset time (the moment during the task when fixation on that cue begins), to reveal how athletes extract and utilize information in the context. A fixation refers to a period during which an individual’s gaze remains on a specific location (Mahanama et al., 2022). Discombe and Cotterill (2015) argue that fixations represent focused attention. Saccades are rapid eye movements that shift gaze between fixation points (Kowler, 2011). These movements typically last only 30–80 ms and allow the brain rapidly sample the visual environment (Discombe and Cotterill, 2015). In addition, smooth pursuits occur when slowly tracking a moving object, and these are not voluntary movements (Kowler, 2011). Other more difficult-to-capture eye movement types, typically detectable only by high-resolution eye trackers, include microsaccades (actions to bring drift back to the center of fixation), tremors (very small eye movements during fixation), and drifts (automatic slow deviations from the center of fixation) (Discombe and Cotterill, 2015). In sport, another widely studied visual strategy variable is the “quiet eye,” which refers to the final fixation or tracking gaze occurring before the initiation of a key action in a motor coordination task (Vickers, 2007, 2009; Vickers et al., 2019). Quiet eye is typically located within 1° to 3° (or less) of visual angle and lasts at least 100 ms (Vickers, 2009; Dalton, 2021). The quiet eye enables athletes to perceive task-relevant environmental cues and construct a motor plan for successful upcoming actions (Dalton, 2021).

Academic interest into why expert athletes outperform novices has grown substantially since the early 21st century (Dalton, 2021). However, establishing who qualifies as a “true expert” remains challenging. For instance, Ericsson’s (1996) “10,000 hours of deliberate practice” theory has been shown to have limitations, contributing to the absence of a clear, unified standard for defining “expert” in academia. Moreover, while superior sports performance is readily observable, the underlying perceptual-cognitive mechanisms are often less visible (Mann et al., 2007). Nevertheless, prior research has primarily compared experts and non-experts based on performance levels (Mann et al., 2007).

Overall, substantial evidence suggests that expert athletes demonstrate superior perceptual cue detection, more efficient eye-movement control, and enhanced attentional processing compared with lower-level performers (Mann et al., 2007; Voss et al., 2010) (Mann et al., 2007; Voss et al., 2010). Specifically, experts demonstrate better visual acuity (Laby et al., 1996; Uchida et al., 2013), stronger visual-perceptual and cognitive abilities (Starkes and Ericsson, 2003; Williams et al., 2011), and superior visual tracking abilities (Vickers and Adolphe, 1997).

However, findings on core quantitative indicators of visual search strategies remain inconsistent. Some studies reported that expert athletes make fewer fixations but maintain longer fixation durations (Vaeyens et al., 2007b; Dalton, 2021), alongside longer quiet eye durations and earlier quiet eye onsets (Vickers, 2009). In contrast, Chia et al. (2017) found that expert badminton players had significantly more fixations, while other studies have reported no significant differences in fixation numbers between experts and novices (Abernethy and Russell, 1987b; Alder et al., 2014).

Furthermore, although Silva et al. (2022) conducted a systematic review and meta-analysis comparing visual search strategies between experts and novices, their work focused on team sports such as soccer (Abellán et al., 2019), volleyball (Afonso et al., 2014), and basketball (Klostermann et al., 2018). Importantly, team and individual sports differ fundamentally in task structure and information processing demands. In team sports, athletes must monitor multiple teammates and opponents simultaneously, with visual search often emphasizing spatial layout, group dynamics, and tactical coordination (BC et al., 2023; Dong et al., 2022). In contrast, individual sports such as tennis (Singer et al., 1996), badminton (Abernethy and Russell, 1987), and table tennis (Piras et al., 2019) typically involve one-on-one confrontational scenarios, where decision-making relies on rapid extraction and processing of ball trajectory, subtle opponent cues, and time pressure (Borysiuk and Waskiewicz, 2008; Takeuchi and Inomata, 2009). Consequently, expert-novice visual search characteristics observed in team sports may not generalise directly to individual sports. To date, however, there remains a lack of a systematic review integrating existing evidence and quantifying expert-novice visual search differences through meta-analysis in individual sports domains such as tennis (Singer et al., 1996), badminton (Abernethy and Russell, 1987), and table tennis (Piras et al., 2019).

Based on the current evidence base, this study aims to address this significant literature gap by conducting a systematic review with meta-analysis of expert–novice differences in visual search behaviour in individual sports. According to prevailing findings in the literature, this study hypothesizes differences between experts and novices, primarily manifesting as experts having fewer fixations but longer fixation durations, with more pronounced quiet eye phenomena. The findings of this study are expected to provide theoretical insights and practical implications for designing new interventions to develop visual search skills in sports training.

## 2 Methods

**Figure 1:**
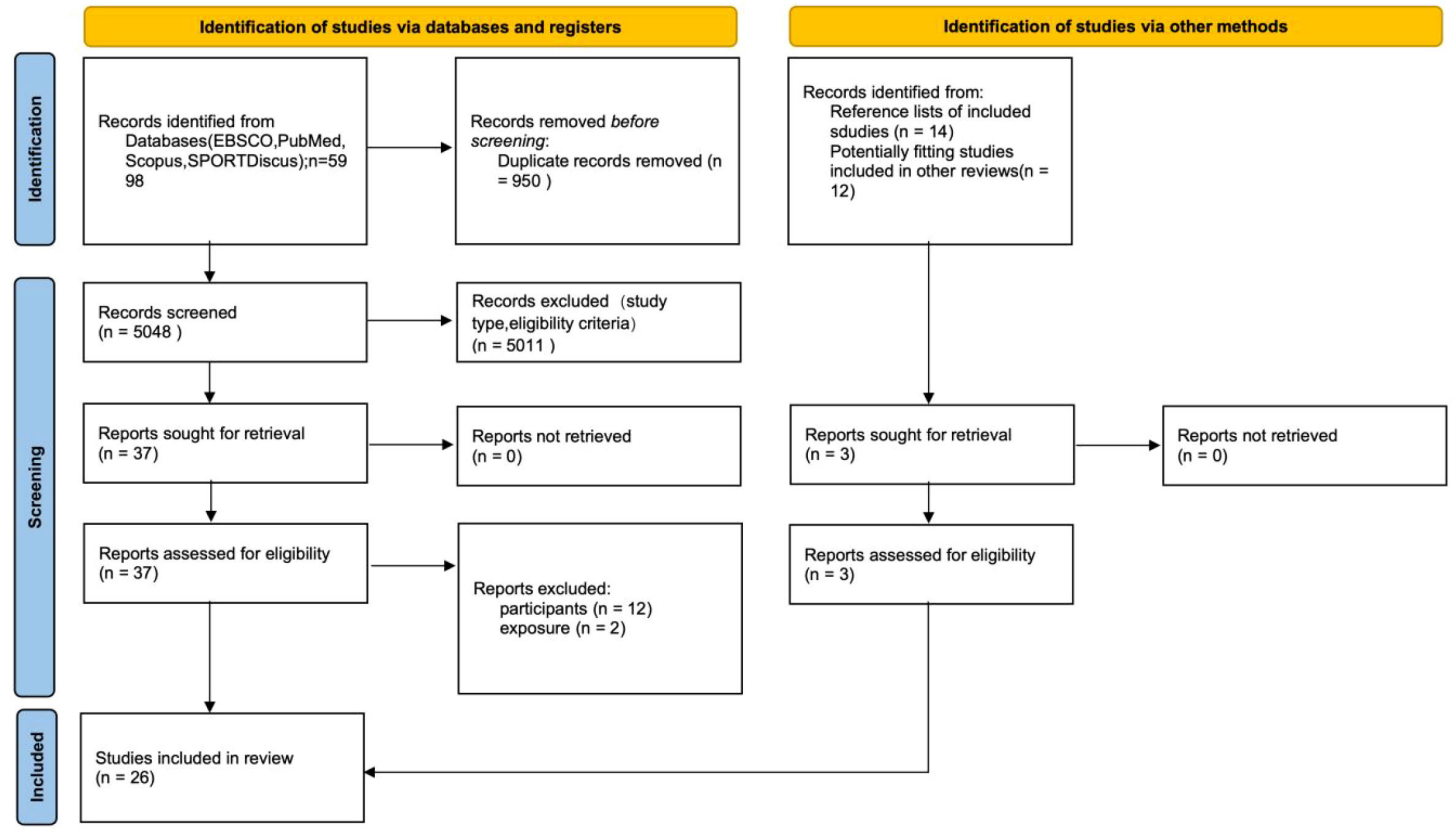
PRISMA 2020 flow diagram.

This systematic review with meta-analysis followed the PRISMA 2020 guidelines (Page et al., 2021) and Cochrane Handbook (Higgins et al., 2019) (**Figure 1**).

### 2.1 Eligibility criteria

Only Studies published in peer-reviewed journals were considered, with no restrictions regarding the date or language. The inclusion criteria were based on the PECOS (Participants, Exposure, Comparator, Outcome, Study design) framework (Morgan et al., 2018).

#### 2.1.1 Participants

Eligible participants were healthy athletes from individual sports who engaged in regular training, regardless of gender, age, or competitive level, and who were pre-classified as experts or novices in the original studies. Studies that classified participants *post hoc* based on test results were excluded. Other classifications preserving the comparative essence were acceptable, such as higher-skilled vs. lower-skilled, high-achievers vs. average performers, or more experienced vs. less experienced.

#### 2.1.2 Exposure

Studies were required to involve exposure to environments that simulated real competitive scenarios (including in “situ” and “film-based” formats), with visual search behaviour had to be assessed using eye-tracking technology suitable for dynamic tasks, such as mobile or head-mounted eye trackers.

#### 2.1.3 Comparator

Studies must include clear between-group comparisons, including at least one expert group and one novice group. If one group was experts, the comparator was required to consist of novice athletes, and vice versa.

#### 2.1.4 Outcomes

Eligible studies must include at least one of the following outcomes: fixation count, fixations per second, fixation location (i.e., areas of interest), number of fixation locations, fixation duration, fixation duration per location, fixation order/sequence, visual field (i.e., total area covered by central and peripheral vision), quiet eye duration, microsaccade and/or saccade amplitude, duration, peak velocity, and acceleration.

#### 2.1.5 Study design

Any study design was eligible including at least one expert group and one novice group.

### 2.2 Information sources

Searches commenced on 1 November, 2025, using the EBSCO (Academic Search Ultimate, APA PsycArticles, and APA PsycInfo), PubMed, Scopus, and SPORTDiscus databases, with no filters applied. After automated searches, reference lists of included studies were manually backward-traced. The preliminary literature list was reviewed by two external experts in the field to verify completeness and identify potential omissions.

Additionally, we screened relevant review articles to identify further eligible primary studies. The search strategy was adjusted by adding “review” in the title field and supplemented in PubMed on 18 November, 2025. Following Higgins et al. (2019) guidelines, we also checked all included studies to check and exclude any formal errata, corrections, addenda, or retractions.

### 2.3 Search strategy

Free-text terms and Boolean operators (AND/OR) were applied to titles or abstracts. No filters or restrictions were used. Some databases only perform wildcard searches (using) *for words with at least four letters, which was considered in our standard search strategy: visual OR visual* OR eye OR ocular OR gaze OR fixation OR eye* OR oculomotor OR decision* OR anticipation* OR “quiet eye” OR saccade* OR “eye task” AND sport* OR athlete* AND expert* OR novice OR skill* OR experience*. The fourth line of the code was applied to full text/all text/any field (depending on the database): “eye-tracking*” OR “eye tracking*” OR “fixation track*” OR “fixation trace*” OR “gaze track*” OR “gaze trace*” OR “oculomotor”.

Full search strategies and details for each database are provided in **Table 1**.

**Table 1:** Full search strategies for each database.

Database	Specificities of the database	Search strategy
EBSCO (Academic Search Ultimate, APA PsycArticles, and APA PsycInfo)	EBSCO does not allow combinations of title and abstract. To avoid multiple internal combinations (eight in total), we decided to use a more open search strategy in this database, with all code lines being open to “All text”.	(vision OR visual* OR eye OR eyes OR gaze OR gazing OR ocular OR oculomotor OR decision* OR anticipa* OR quiet eye OR saccad* OR eye task) AND (sport* OR athlete*) AND (expert* OR novice OR skill* OR experience*) AND (eye-track OR eye track OR fixation track* OR fixation-track* OR gaze-track* OR gaze track* OR eye movement)
PubMed	Nothing to report.	(((Vision[Title/Abstract] OR visual* [Title/Abstract] OR eye[Title/Abstract] OR eyes[Title/ Abstract] OR gaze[Title/Abstract] OR gazing[Title/Abstract] OR ocular[Title/Abstract] OR oculomotor[Title/Abstract] OR decision* [Title/Abstract] OR anticipa* [Title/Abstract] OR “quiet eye” [Title/Abstract] OR saccad* [Title/Abstract] OR “eye task”[Title/Abstract]) AND (Sport* [Title/Abstract] OR athlet*[Title/Abstract])) AND (expert* [Title/Abstract] OR novice[Title/Abstract] OR skill*[Title/Abstract] OR experience*[Title/Abstract])) AND (“eye-track*” OR “eye track*” OR “fixation track*” OR “fixation-track*” OR “gaze-track*” OR “gaze track*” OR “eye movement”)
Scopus	In Scopus, the search for title or abstract also includes keywords.	(TITLE-ABS-KEY (vision OR visual* OR eye OR eyes OR gaze OR gazing OR ocular OR oculomotor OR decision* OR anticipa* OR “quiet eye” OR saccad* OR “eye task”) AND TITLE-ABS-KEY (sport* OR athlet*) AND TITLE-ABS-KEY (expert* OR novice OR skill* OR experience*) AND ALL (“eye-track*” OR “eye track*” OR “fixation track*” OR “fixation-track*” OR “gaze-track*” OR “gaze track*” OR “eye movement”)
SPORTDiscus	SPORTDiscus does not allow combinations of title and abstract. To avoid multiple internal combinations (eight in total), we decided to use a more open search strategy in this database, with all code lines being open to “All text”.	TX (Vision OR visual* OR eye OR eyes OR gaze OR gazing OR ocular OR oculomotor OR decision* OR anticipa* OR “quiet eye” OR saccad* OR “eye task”) AND TX (Sport* OR athlet*) AND TX (expert* OR novice OR skill* OR experience*) AND TX (“eye-track*” OR “eye track*” OR “fixation track*” OR “fixation-track*” OR “gaze-track*” OR “gaze track*” OR “eye movement”)

The asterisk (*) is a wildcard that means "any letters or characters" after the word.

### 2.4 Selection process

QQ and YL independently screened each record, with discrepancies resolved by DX. Duplicates were manually removed.

### 2.5 Data collection process

QQ and YL independently collected data, with discrepancies arbitrated by DX. No automation tools were used.

### 2.6 Data management

Data extraction followed a structured framework coverung primary, secondary, and additional variables. Primary outcomes captured key visual-search metrics, including fixation count and frequency, fixation locations (with counts and durations per location), and overall fixation duration and quiet eye duration.Oculomotor parameters were also extracted, including visual field measures and (micro)saccade characteristics (amplitude, duration, peak velocity, and acceleration).Secondary outcomes focused on performance-based metrics, including task response time, decision-making efficacy, and motor response accuracy. Additional variables were extracted across three domains: (1) experiment-related factors, such as setting (video-based, *in situ*, or both), projection area (for video-based studies), exposure descriptions, eye-tracking specifications (model, sampling rate, resolution, and noise), fixation/saccade definitions, and calibration frequency; (2) sample-related factors, including the specific sport, competitive level (or expertise indicators), sample size, age, gender, and years of practice; and (3) administrative factors, comprising study location, competing interests, and funding sources.

### 2.7 Risk of bias assessment of studies

**Figure 2:**
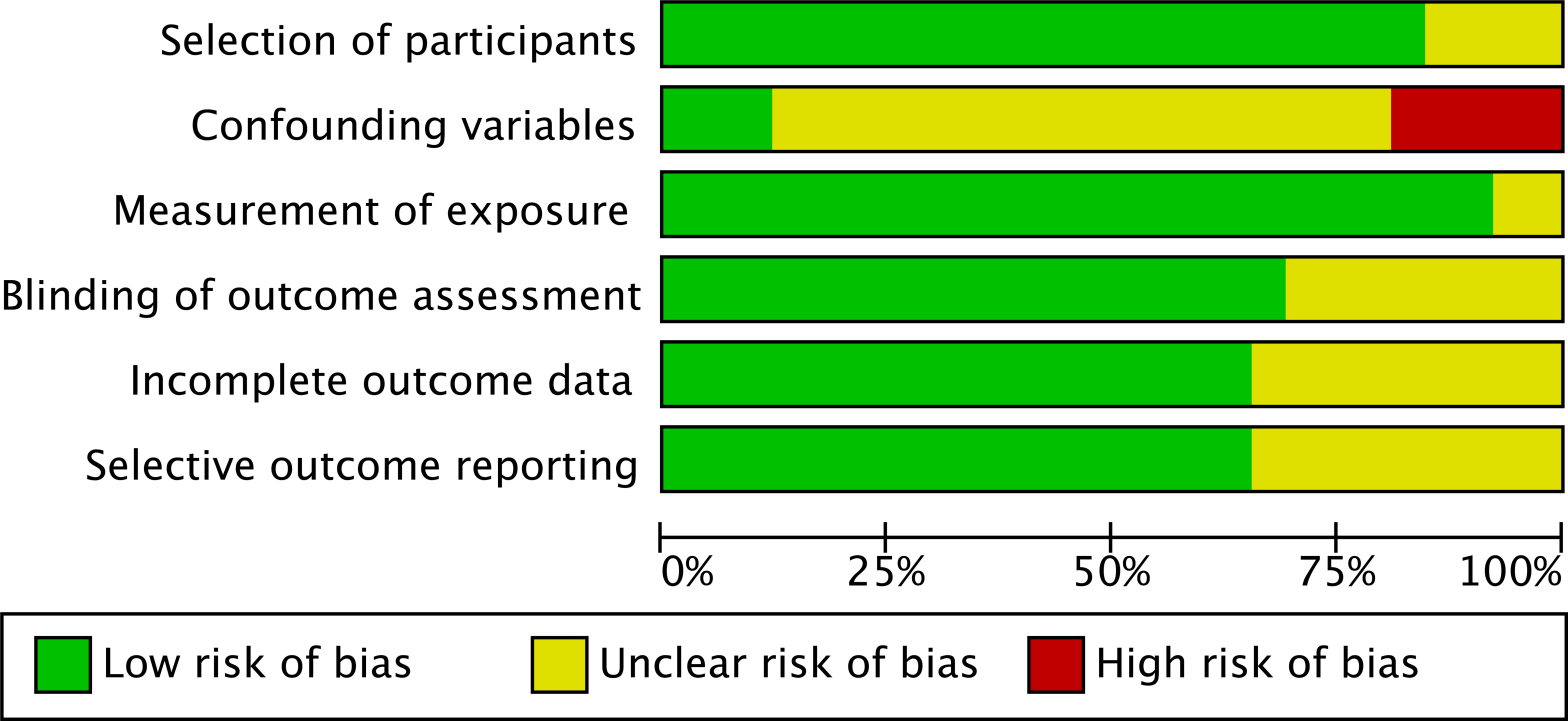
Risk of bias analysis of included studies.

Since included studies used non-randomized designs (comparing experts and novices), the term “exposure” more accurately reflected study characteristics than “intervention.” Thus, risk of bias was assessed using Cochrane’s RoBANS tool (Park et al., 2011), Assessment dimensions included (i) participant selection; (ii) confounding variables; (iii) measurement of exposure; (iv) blinding of outcome assessment; (v) incomplete outcome data; (vi) selective outcome reporting. Judgments for each study on these dimensions (low risk/high risk/unclear) and supporting rationales were entered into Review Manager 5 (RevMan 5), which generated a risk of bias summary table and graph (**Figure 2**).

### 2.8 Data synthesis

For continuous outcomes (e.g., fixation duration, fixation duration per AOI, visual field indicators, quiet eye duration, microsaccades, and saccade amplitude/duration/peak velocity/acceleration), meta-analyses were conducted using Review Manager (The Cochrane Collaboration, 2020) to generate forest plots when at least three sufficiently homogeneous studies reported the same outcome (Claudino et al., 2021). Effect sizes for continuous outcomes were pooled using standardized mean differences (SMDs), which RevMan implements as Hedges’ g, calculated based on means, standard deviations, and sample sizes of expert and novice groups.

For count or frequency-related variables (e.g., fixation count, fixations per second), ourcomes reported as mean ± standard deviation were treated as continuous outcomes and pooled using SMDs (Hedges’ g). When studies reported ratios or other effect sizes (e.g., rate ratios/odds ratios), they were converted into comparable formats using Cochrane Handbook (Higgins et al., 2019) and entered via Generic Inverse Variance in RevMan as “effect size and its standard error.” For studies reporting standard error (SE) only, group standard deviations were calculated as SD = SE × √n per Cochrane Handbook before data entry into RevMan.

Meta-analyses were conducted using Review Manager (RevMan 5). Given expected heterogeneity in task contexts, participant characteristics, and measurement methods among included studies, a random-effects model with inverse variance method was used (Inverse Variance, Random; displayed as “IV, Random” in forest plots). Continuous outcomes were summarized as standardized mean differences (SMDs; forest plot header as “Std. Mean Difference, IV, Random, 95% CI”) with 95% confidence intervals (95% CI). Under the random-effects model, study weights are determined by the precision of estimates, typically proportional to the inverse of variance (smaller standard error, larger weight). Overall effect significance was tested using Z-test, reported in RevMan output as “Test for overall effect: Z = : P = :”. Between-study heterogeneity was assessed using RevMan outputs of τ², χ² (Q), and I², reported as “Heterogeneity: Tau² = :; Chi² = : df = : (P = :); I² = :%”; I² ≈ 25%, 50%, 75% are generally interpreted as low, moderate, and high heterogeneity, respectively. Effect size magnitude followed Hopkins et al.’s grading: <0.2 trivial; 0.2–0.6 small; >0.6–1.2 moderate; >1.2–2.0 large; >2.0–4.0 very large; >4.0 extremely large.

Nominal variables (i.e., fixation locations/AOIs) were presented as raw frequencies (n) and percentages (%) of total fixations. Based on this study’s AOI classification scheme, fixation locations were grouped into eight categories: head, chest, pelvis, abdomen, right shoulder, left shoulder, fore arm, and hind arm. Contingency analysis was performed using SPSS (Version 27), with chi-square test (χ²) comparing distribution differences between expert and novice groups based on raw frequencies. Effect size was calculated using Cramér’s V, interpreted by correlation strength: very weak (0–0.19), weak (0.20–0.39), moderate (0.40–0.59), strong (0.60–0.79), and very strong (0.80–1.00). To identify the primary contributors to any group differences, adjusted standardized residuals were examined; when |residual| > 1.96, the cell’s observed frequency significantly exceeds (positive residual) or falls below (negative residual) the expected frequency under the null hypothesis. When more than 20% of cells in the contingency table had expected counts <5, a Monte Carlo correction was applied to obtain more robust significance testing.

Subgroup and/or sensitivity analyses were conducted where by the number of studies per outcome premitted. Pre-specified grouping/moderating factors included: (i) gender; (ii) sport type; (iii) age group; (iv) risk of bias level; and (v) experimental setting (in situ/field vs. film/video-based). Meta-analyses and subgroup analyses were performed in Review Manager (RevMan 5). Sensitivity analyses (e.g., excluding studies one-by-one, changing pooling models) were conducted within RevMan 5 to test the robustness of pooled effects. Analyses of categorical variables (e.g., contingency analysis for fixation location distributions, chi-square tests, and effect size calculations) were completed in IBM SPSS (Version 27). Statistical significance was set at p < 0.05.

### 2.9 Risk of reporting bias

For continuous outcomes, publication bias was assessed when at least 10 studies were available for a given outcome, in line with methodological recommendations (Sterne et al., 2011), Egger’s regression test was performed in Stata 18 and/or funnel plots were generated in Review Manager (RevMan 5) for visual inspection to explore potential publication bias. If test results met the preset significance levels threshold, bias risk was considered. To further evaluate the impact of potential missing studies on pooled effects, sensitivity analysis was conducted in Stata (18) using Duval and Tweedie’s trim-and-fill method (Shi and Lin, 2019), with the number of estimated missing studies as the default fill value, comparing changes in pooled effect sizes before and after adjustment to test conclusion robustness.

## 3 Results

### 3.1 Study selection

The search retrieved 5,998 records, of which 950 were duplicates. Titles and abstracts of the remaining 5,048 records were screened, and 37 articles were retrieved full-text assessment (**Figure 1**).

Of these, 12 studies were excluded for lacking expert and novice groups (Bagot et al., 2023; Hausegger et al., 2019; Kim et al., 2019; Krabben et al., 2021, 2022; Milazzo et al., 2016a; Mitchell et al., 2020; Ryu et al., 2013; Shinkai et al., 2022, 2025; Singer et al., 1998; Witkowski et al., 2019), and 2 were excluded for lacking eye trackers (Muiños and Ballesteros, 2013; Abernethy and Russell, 1987).

**Tables 2**–**4** summarized details on the 23 included studies in our review (Abernethy and Russell, 1987; Abernethy, 1990; Alder et al., 2014; Bandow and Witte, 2021, 2020; Chia et al., 2017; Guo et al., 2024; Hagemann et al., 2010; Kato, 2020; Lin et al., 2021; Milazzo et al., 2016a; Murray and Hunfalvay, 2017; Nibbeling et al., 2012; Piras et al., 2014; Piras et al., 2019; Rauniyar and Srivastava, 2025; Ripoll et al., 1995; Sáenz-Moncaleano et al., 2018; Shirmehenji et al., 2019; Singer et al., 1996; Ward et al., 2002; Williams and Elliott, 1999; Witkowski et al., 2021). Manual searching of reference lists identified 14 potentially relevant titles, 13 of which were already in our database search. The remaining 1 underwent full-text analysis and met all inclusion criteria (Takeuchi and Inomata, 2009). In addtion, two relevant reviews on the topic were identified, which yielded further 12 potential eligible titles. 10 of these were already in our initial search, but 2 were not. Full-text analysis indicated they met all inclusion criteria (Goh et al., 2018; Goulet et al., 1989).

**Table 2:** Study characteristics and main results.

Study	Sample				Years of practice and competitive level	
	N	Sex	Grouping (Expert vs. Nonexpert)	Age	Years of practice	Competitive level
Abernethy and Russell (1987)	31	M=23, F=8	Expert (N= 15)Novice(N = 16)	Expert: 18–32Novice: 18–29	Expert: Participants in the VIIth Commonwealth GamesNovice: Undergraduate students	Expert and Novice
Abernethy (1990)	E1:32E2:8	E1:M=16 ;F=16E2:M=4 ;F=4	E1:Expert(N=15);Novice(N=17)E2:Expert(N=4);Novice(N=4)	E1:Expert= 17-45(M= 23.3);Novice=17-28(M= 20.2)E2:Expert= 19-30;Novice=19-29	Not reported	Expert and Novice
Alder et al. (2014)	16	Not reported	Experts = 8,Novices = 8	Experts: 28.9 ± 3.1 Novices: 18.5 ± 1.1	Experts: ≥10 Novices: no structured badminton training or competition	Experts and Novices
Bandow and Witte (2020)	26	M=15, F=11	Skilled (N=16)Less-skilled (N=10)	Skilled:22.5±5.3Less-skilled:15.8±2.4	Skilled: 14.7 ±3 Less-skilled: 6.8 ±2	Skilled and Less-skilled
Bandow and Witte (2021)	11	M=8, F=3	Skilled (N=6)Less-skilled (N=5)	Skilled: 15.5 ±1.8Less-skilled: 14.6±1.1	Skilled: 9.1± 2.3Less-skilled: 8.7 ±2.1	Skilled and Less-skilled
Chia et al. (2017)	24	M=12, F=12	Skilled (N=12)Less-skilled (N=12)	Skilled: 22.4±1.38 Less-skilled: 25.5±3.45	Skilled: 9.25±3.25 Less-skilled: None	Skilled and Less-skilled
Goh et al. (2018)	21	Not reported	Elite = (N=11),Sub-elite = (N=10)	Elite: 21.4 ± 3.3 yrsSub-elite: 13.7 ± 1.2	Elite: 8.6 ± 2.0 Sub-elite: 5.1 ± 0.9	Elite and Sub-elite
Goulet et al. (1989)	E1: 29E2: 20	E1:Experts: M = 7, F = 8,Novices: M = 8, F = 6E2: Not reported	Experts vs Novices	E1:Experts: M = 22.3,Novices: M = 21.6 E2:Experts: M = 22.1,Novices: M = 21.2	Experts: ranked among top 40 players in Quebec or former ranked players serving as coachesNovices: undergraduate physical education students with no elite competitive background	Experts and Novices
Guo et al. (2024)	40	M=24, F=16	Expert (N=20)Novice (N=20)	Expert: M=21.03±2.30,F=20.21±1.82 Novice: M=19.15±1.41 , F=19.43±0.83	Expert: 6.07±1.03 Novice: 2+	Expert and Novice
Hagemann et al. (2010)	62	M=39, F=23	Expert (N=15),Advanced (N=15),Novice (N=32)	Expert: 20.36±4.63,Advanced: 24.25±7.18,Novice: 24.70±2.66	Expert: 12.20±4.63 Advanced: 12.09±5.12,Novice: 0	Expert,Advanced and Novice
Kato (2020)	20	M=20, F=0	Expert(N=10)Novice(N=10)Shihan(N=1)	Expert=20.4±1.4Novice=20.8±1.3 Shihan=65	Expert=13.7±3.2, Novice=3.9±1.4	Expert,Shihann and Novice
Lin et al. (2021)	10	M=10, F=0	Expert (N=5) Novice (N=5)	Expert: 20.6 ± 1.1Novice: 20.0 ± 0.4	Expert: 10.2 ± 1.1 Novice: 1 ± 0.5	Expert and Novice
Milazzo et al. (2016a)	28	Not specified	Expert (N=14)Novice (N=14)	Expert: 25.6 ±2.5Novice: 27.4 ±5.5	Expert: 15.9 ±2.2Novice: 1.6 ±0.7	Expert and Novice
Murray and Hunfalvay (2017)	43	M=21, F=22	Elite (n=21) Non-elite (n=22)	Mean age: 23.63 ± 3.59	Elite: 16.74 ± 4.52 Non-elite: Varies	Elite and Non-elite
Nibbeling et al. (2012)	20	M=16, F=4	Experts = 11Novices = 9	Experts: 34.2 ± 9.6Novices: 22.9 ± 1.7	Experts: 11 ± 5 Novices: No dart experience	Experts and Novices
Piras et al. (2014)	20	M=14, F=6	Expert (N=9)Nonexpert (N=11)	Expert: 26.89 ± 6.68Nonexpert: 23.64 ± 1.80	Expert: 16 years (national competition)Nonexpert: No competitive experience	Expert and Nonexpert
Piras et al. (2019)	25	M = 25, F = 0	Experts = 10,Novices = 15	Experts: 23.10 ± 7.58 Novices: 26.8 ± 3.73	Experts: ~10 ± 2.60 Novices: no reported professional table tennis experience	Experts and Novices
Rauniyar and Srivastava (2025)	20	M=20, F=0	Combat Athletes (N=10), Non-athletes (N=10)	Mean age: 21.50 ± 2.80	Combat Athletes: 6 months of active training,Non-athletes: No athletic background	Combat Athletes and Non-athletes
Ripoll et al. (1995)	18	Not specified	Expert (N=6)Intermediate (N=6)Novice (N=6)	Expert: 27.3Intermediate: 23.8 Novice: 26.3	Expert: National team boxersIntermediate: training once a weekNovice: more than one year ofpractice	Expert,Intermediate and Novice
Sáenz-Moncaleano et al. (2018)	21	M=10; F=11	High level:(N=10)medium level: (N=11)	High level: 21.6±2.12medium level: 22.45±3.50	High level: over three more years; medium level: once or twice per week	Not reported
Shirmehenji et al. (2015)	22	M=0,F=22	Skilled (N=10)Non-skilled (N=12)	Skilled: 25.2 ± 4.04Non-skilled: 22.25 ± 2.80	Skilled: exact number not reported; Non-skilled: completed Badminton I course only, no competition experience	Skilled and Non-skilled
Singer et al. (1996)	60	M=30, F=30	Highly-skilled (N=30),Beginners (N=30)	Not specified	Highly-skilled: Not specified,Beginners: Not specified	Highly-skilled and Beginners
Takeuchi and Inomata (2009)	14	M=14, F=0	Expert (N=7)Nonexpert (N=7)	Expert:19.4± 1.7Nonexpert:22.4± 2.8	Expert: Not specified,Nonexpert: Not specified	Expert and Nonexpert
Ward et al. (2002)	16	Not reported	Experienced(N=8)Inexperienced(N=8)	Experienced: 23.0±7.3Inexperienced:27.2±4.4	Experienced: 11.9 ±4.7Inexperienced: 3.8 ± 1.0	Experienced and Inexperienced
Williams and Elliott (1999)	16	M=16,F=0	Expert(N=8)Novice(N=8)	Experts: 22.0±2.3 Novices: 26.3±4.6	Experts: ≥3 years formal karate training (5.6 ±2.3)Novices: no formal training	Experts and Novices
Witkowski et al. (2021)	19	M=9, F=10	Expert (N=9)Beginner (N=10)	Expert: 18-31 Beginner: 16-19	Expert: ≥ 10 Beginner: ≤ 4 years	Expert and Beginner

**Table 3:** Study characteristics and main results.

Study	Study location and sport		Primary outcomes and second outcomes		Exposure description
Author and Year	Study location	Sport	Primary outcomes	Second outcomes	Exposure description
Abernethy and Russell (1987)	Laboratory	badminton	Decision making	prediction erroranticipatory capabilityFixation locations following screen centre fixationsFixation locations preceding and following trunk fixationsFixation locations preceding and following head fixationsFixation locations preceding and following racquet fixationsFixation locations preceding and following shuttle fixationsFinal fixation location occurrences	Film task with eye movement recording of expert and novice badminton players; Experiment to assess visual search process and prediction ability
Abernethy (1990)	Laboratory	Squash	Decision making	E1:Accuracy in predicting stroke direction and forcemean fixation duration search rate Mean trial time per location / % E2:mean distribution of fixation points mean fixation durationsearch rate	E1: Film task with eye movement recording of expert and novice squash players; Experiment E2: Field task recording eye movement data and prediction ability of athletes
Alder et al. (2014)	Laboratory	Badminton	Decision making	response accuracyResponse errorsnumber of visual fixationsMean duration of fixationfixation locations	Section 1: Kinematic analysis 4 elite players performed long/short serves; 28 markers recorded with motion capture; discriminating cues found in wrist/arm/racket during execution phase.Section 2: Temporal occlusion anticipation test8 experts & 8 novices viewed serves filmed from opponent perspective; occlusion at −40 ms / contact / +40 ms; performed anticipation + eye tracking.
Bandow and Witte (2021)	Laboratory	Karate	visual behavior	mean number of fixationsFixation duration	Eye movement data recorded during the last 2000 ms before executing an attack
Bandow and Witte (2020)	Laboratory	Karate	visual behavior	Number of relative FixationsFixation durationFixation Locations	View video-based gyaku tsuki and mawashi geri attacks with 4 occlusion conditions (3 specific body parts occluded + non-occluded), each sequence repeated 3 times; record gaze behaviour of skilled and less-skilled athletes
Chia et al. (2017)	indoor air-conditioned badminton court	Badminton	visual behavior	mean number of fixationsNumber of fixation locationsGaze distributionQuiet eye durationQuiet eye location	Recording eye movement data during the badminton serve and analyzing visual search behavior
Goh et al. (2018)	international-standard bowling lanes in Singapore	Ten-pin bowling	visual behavior	Number of fixation locationsfixated frequentlyFixation timeFixation LocationsNumber of fixationsQuiet eye locationmovement-QE onsetmovement-QE duration	Participants: 11 elite vs 10 sub-elite bowlers; tasks performed under low-anxiety (LA) and high-anxiety (HA) conditions.Eye movements recorded using Dikablis mobile eye tracker during pre-movement and movement (five-step approach) phases.AOIs included dots, arrows, breakpoint, and pin deck; QE analysed separately for pre-movement and movement.
Goulet et al. (1989)	Laboratory	Tennis	Decision making	Number of Correct ResponsesPercentage of correct responsesdecision timeNumber of fixations Scanpaths	Experiment 1: Eye-movement recordingParticipants viewed 16-mm films of flat, topspin, and sliced serves (right- and left-handed servers); eye movements recorded to analyse fixation number, fixation locations, and scanpaths across ritual, preparatory, and execution phases.Experiment 2: Temporal occlusion taskServe sequences occluded at different phases (preparatory only; preparatory + early execution; up to contact; ritual to contact; full vision). Measured decision time and accuracy in identifying serve type.
Guo et al. (2024)	a specific park	Orienteering	Decision making	correct rateresponse rateon the figure:fixation time Number of fixationson the real scene:Fixation timefixation hotspot mapfixation trajectory diagram	Conducting image recognition task tests with eye-tracking technology in real-world orienteering environments
Hagemann et al. (2010)	Laboratory	Fencing	Decision making	Prediction performanceFixation timeNumber of fixations	The subjects watched 405 short video clips of fencing attacks and their eye movement data were recorded
Kato (2020)	Laboratory	Kendo	Decision making	Number of attempts Success rate Number of blocked attacksNumber of offensive techniques;Number of counter techniques;Number of defensive techniques ;Number of awarded points ;The mean number of fixationsmean fixation durationmean number of fixation locationsPercentage viewing time;	in situ conditions, eye movement data and match performance data were recorded.
Lin et al. (2021)	Laboratory	Tennis	Visual behavior	mean fixation duration	Eye movement data recorded during the video watching of tennis serves using eye-tracking technology
Milazzo et al. (2016a)	Laboratory	Karate	Decision making	Decision TimeDecision AccuracyAwareness of PatternMean fixation durationNumber of visual fixationsNumber of fixation locationsPercentage viewing timeVerbal Reports	Field task recording eye movement data and decision-making behavior of athletes
Murray and Hunfalvay (2017)	Laboratory	Tennis	visual behavior	fixation durationNumber of visual fixationsfixation locations	Participants viewed 18 professional tennis serves (flat, slice, kick × 3 directions)
Nibbeling et al. (2012)	indoor climbing wall	Dart throwing	Decision making	Anxiety scoresHeart rateDart scoresDart timesResponse ratePercentage of correct countsInvested mental effortPerceived exertionScan ratioTotal fixation durationdrift durationDuration of the final fixation on bulls eyeFinal fixation onsetFinal fixation offset	Dart throwing under low/high anxiety; single-task vs dual-task (counting backwards in 3s).
Piras et al. (2014)	Laboratory	Judo	visual behavior	fixation durationNumber of visual fixationsFixation transitionsNumber of fixation locationsPercentage viewing time	Recording eye movement data during real judo sparring to analyze visual search behavior
Piras et al. (2019)	Laboratory	Table tennis	visual behavior	Response accuracyKey-press response timeMean number of fixationsmean fixation durationsIAs fixationsSaccade characteristicsMicrosaccade characteristicsMicrosaccades direction	Participants viewed 20 stroke videos (10 forehand topspin; 10 backhand drive) filmed from first-person perspective; predicted ball-landing side while eye movements were recorded. Dynamic interest areas (IAs): head, trunk, hand-racket, ball; all updated frame-by-frame.
Rauniyar and Srivastava (2025)	Laboratory	Mixed Martial Arts, Boxing	visual behavior	fixation durationTime to First Fixation	Eye movement data recorded while participants watche
Ripoll et al. (1995)	Laboratory	French Boxing	Decision making	reaction timeAccuracy of motor responsesEfficacy of decision-makingNumber of fixationsFixation locationTotal fixation durationMean duration fixationScan-paths	E1: In a film-based task, the reaction time and accuracy of three groups of athletes were recorded. E2: In a film-based task, the eye movement data of the athletes were recorded.
Sáenz-Moncaleano et al. (2018)	Laboratory	Tennis	visual behavior	Mean Fixation DurationsTotal Sum of F/TPrebounce FixationsBounce FixationsPostbounce FixationsQEOnsetQE Duration	Participants returned 40 serves in 4 serve locations while wearing a mobile eye tracker. The ball's flight path was deconstructed into 3 distinct locations (i.e., ball before bouncing on surface, the bounce area, and ball after bouncing on surface), and gaze behaviors along with quiet-eye (QE) onset and durations were recorded
Shirmehenji et al. (2015)	Laboratory	Badminton	Decision making	Anticipation behavior Accuracy percentage of anticipation behaviorNumber of visual fixationsfixation duration	Recording eye movement behavior of players while watching long serve clips and evaluating anticipation accuracy
Singer et al. (1996)	Laboratory	Tennis	visual behavior	number of fixationstotal fixation durations	Viewing simulated tennis serves and ground strokes to assess visual search behavior and anticipation
Takeuchi and Inomata (2009)	Laboratory	Baseball	visual behavior	Time of pitcher’s motion Number of visual fixationsNumber of areas fixatedthe mean percentage of the viewingNumber of strike ballsAverage velocities of pitchesthe accuracy of pushing the buttonaverage velocities of pushing the button	Using eye-tracking to record the visual search behavior of players during the pitcher’s motion and measuring the accuracy of swing judgments
Ward et al. (2002)	Laboratory	Tennis	visual behavior	percentage ofviewing timeSearch rateSearch order	Two viewing conditions: normal film & point-light biological motion; participants viewed forehand/backhand ground strokes and made anticipatory stepping responses via pressure pads
Williams and Elliott (1999)	Laboratory	Karate	Decision making	Viewing time (VT) Response accuracy (RA)Fixation durationNumber of fixationsNumber of fixation locations	10 karate techniques filmed from front view; high vs low anxiety conditions; anticipation task predicting opponent’s attack
Witkowski et al. (2021)	on a piste in a well-lit fencing hall	Fencing	visual behavior	Dwell time (%)Average fixationGlance countFixation count	Recording eye movement data during fencing duels to analyze visual search strategies

**Table 4:** Study characteristics and main results.

Study	Experimental design and experimental setting (film-based; in situation; both)		Eye tracker specifications	Study definition of fixation / saccades / microsaccades & way of calibration	Frequency of calibration
	Experimental design	Experimental setting (film-based; in situ; both)			
Abernethy and Russell (1987)	Between groups comparison	Film-based	Polymetric Mobile V0165 eye-movement recorder with ≈1° accuracy within ±10° horizontally and vertically;	Fixation definition: a fixation was any case where the eye mark remained stationary for ≥ 3 video frames (120 ms) at 25 Hz sampling.	This calibration was checked at regular intervals throughout the duration of the experiment
Abernethy (1990)	Between groups comparison	Film-based and In situation	NAC EMR-V eye-mark recorder with ± 1° accuracy; field of view: 60° horizontally, 45° vertically	Fixation definition : Fixations were operationally defined as any case where the eye mark remained stationary for a minimum of 120 ms (3 frames)Calibration : calibrated for both position and linearity with the use of a standard projection slide	This calibration was checked at regular intervals throughout the duration of the experiment
Alder et al. (2014)	Between groups comparison	Film-based	ASL MobileEye (monocular); head-mounted; samples at 25 fps; computes gaze via pupil–cornea vector; eye-video coded frame-by-frame in Adobe Premier Pro.	Fixation: gaze within 3° visual angle for ≥ 120 ms Final fixation: last fixation prior to occlusion. Fixation-location categories: racket, wrist, shuttle, other (only kinematic-relevant AOIs analyzed). Calibration: 6-point calibration on still frame (head, racket head, left foot, shuttle, partner’s head & racket). Participants adopted return-stance during calibration.	Calibration checked after 10 familiarization trials, and again between the two blocks (two blocks × 36 trials).
Bandow and Witte (2020)	Mixed design	Film-based	SensoMotoric Instruments Eye Tracking Glasses (ETG); 30 Hz sampling rate; 0.1° visual resolution; 0.5° precision; visual range: 80° horizontal, 60° vertical / SensoMotoric Instruments	Fixation definition: fixations ≥ 100 ms (defined by SMI BeGaze software)Calibration: three-point calibration before experiment; calibration check after each attack; recalibrate when necessary	Before each examination + after each sequence
Bandow and Witte (2021)	Mixed design	In situation	SMI Eye-Tracking Glasses (ETG); binocular; sampling rate 30 Hz; resolution 0.1°; accuracy 0.5°; visual range 80°(horizontal) × 60°(vertical); scene camera 24 Hz, 1280×960 px	Fixation: gaze staying within 3° of visual angle on an opponent’s body part for ≥ 99 ms Calibration: three-point calibration before/after each trial; triangle targets behind defender; recalibrate whenever drift detected	Before and after each trial, plus calibration check after every attack with recalibration if needed
Chia et al. (2017)	Between-groups comparison	In situation	Applied Science Laboratories (ASL) Mobile Eye-XG system; video-based monocular corneal-reflection eye tracker; sampling rate 30 Hz; accuracy 0.5–1° of visual angle; visual range 60° (horizontal) × 40° (vertical).	Fixation: gaze remaining stationary at a specific location for ≥100 ms (≥4 video frames) within ≤3° visual angle. Calibration: nine-point grid calibration at the side of the court before testing	Initial nine-point calibration once before the session; calibration check before every trial with recalibration as needed.
Goh et al. (2018)	2 × 2 mixed design	In situation	Dikablis mobile eye tracker (Ergoneers GmbH); head-mounted; accuracy ±0.5° visual angle; sampling rate 25 Hz; eye camera + scene camera synchronised; data processed using D-Lab Control software with AOI marker boards.	Fixation: gaze at the same location for ≥ 120 ms (≥3 frames at 25 Hz), automatically identified by D-Lab Control. Quiet Eye (QE): final fixation ≥120 ms before movement initiation (pre-movement QE) or before ball release (movement QE); QE duration and onset manually coded. Calibration: initial calibration on four wall points (~1 m distance)	Calibration checked before each anxiety condition (LA and HA); recalibration performed when required; additional calibration after familiarisation.
Goulet et al. (1989)	Between-groups comparison	Film-based	NAC eye-movement recorder (Model V); accuracy 1°; horizontal/vertical ranges ±25°/±20°; corneal reflection technique; connected to Panasonic	Fixation definition: eye mark remained stationary for ≥ 4 frames (133 ms); eye movements analyzed frame-by-frame; video sampling rate 30 Hz (30 fps). Calibration / adjustment: NAC was adjusted to the subject after familiarization	Not reported
Guo et al. (2024)	Single-factor design	In situation	German SMI Eye-tracking glasses, model ETG 2w; sampling frequency 60 Hz (binocular); tracking resolution <0.1°; accuracy 0.5°; tracking distance >40 cm; visual range 80° (horizontal) × 60°	Fixation: saccade counts = rapid eye movements between fixations; saccade frequency = saccades per time unit.Calibration: prior to the formal experiment, eye tracker was calibrated to each participant’s viewing position to ensure accuracy;	Calibration performed twice: once before the practice phase and once immediately prior to the formal test phase.
Hagemann et al. (2010)	Between groups comparison	Film-based	Head-mounted Eyelink II system, with a frequency of 500 hertz	Calibration is carried out after the ninth point calibration and then calibration verification is conducted.	Calibration once before experiment; recalibration performed whenever drift was detected.
Kato (2020)	Within-subjects design	In situation	a lightweight eye movement registration system EMR-9;sampling rate60 Hz;Its precision <0.1°	Fixation definition :Fixation was defined as the period when the eye remained stationary within 1 degree of movement tolerance for a period >99 MS (three video framesCalibration:the system was calibrated and validated with a nine-point reference grid presented ∼2.5 m in front of the fighters	Accuracy was checked before and after each trial, with recalibration performed if necessary.
Lin et al. (2021)	Between-groups comparison	Film-based	Mobile Eye ASL ME XG portable eye tracker; monocular corneal-reflection system; sampling rate 30 Hz; accuracy ≤0.5°; resolution ≤0.1°; horizontal FOV 50°, vertical <40°; gaze data processed with GazeTracker software.	Calibration: computer-assisted pupil correction before the test; required calibration accuracy >80%	Calibration once before the experiment and recalibrated after 3 practice serves; additional recalibration occurred anytime drift or equipment disturbance was detected.
Milazzo et al. (2016a)	Between-groups comparison	In situation	SMI Eye-Tracking Glasses 2.0; binocular sampling 60 Hz; accuracy 0.5°;	Fixation: location-based identification from frame-by-frame coding of gaze ; fixation duration, fixation count, and number of fixation locations used as search-rate indicators. Calibration: 1-point calibration on opponent before experiment	Before each examination + after each sequence
Murray and Hunfalvay (2017)	Between-groups comparison	Film-based	ERICA eye-tracking system; infrared corneal reflection; sampling rate 60 Hz; accuracy ±0.5° visual angle; 21-inch monitor at 60 Hz; viewing distance 90 cm; field of view 18.72° × 24.28°	Fixation defined as eye stable ≥100 ms with <1° of visual movement. Calibration: automatic one-point calibration, followed by 16-point screen calibration;	Calibration checked before familiarization trials and again before the full test video; recalibration applied if drift occurred.
Nibbeling et al. (2012)	2×2×2 mixed design	In situation	the mobile eye tracker (Applied Science Laboratories, Bedford, USA)	Fixation: gaze on same point ≥3 frames (3 × 33.33 ms ≈ 99.99 ms) counted as a fixation. Four gaze categories: bulls-eye / board / other / drift (gaze leaving bulls-eye and returning). Calibration: via Quiet Eye Solutions software using hand-clap synchronization.	Recalibrated before each block (i.e., before each set of 6 throws); additional drift monitoring during trials.
Piras et al. (2014)	Between-groups comparison	In situation	EyeLink II (SR Research Ltd.); binocular system with dual eye cameras;	Fixation: gaze stable within ≤1° visual angle for ≥3 frames (≈99 ms). Saccade: gaze shift ≥2 frames (≈66 ms). Calibration: 9-point grid at ~80 cm + 3-D depth calibration;	Calibration before experiment + drift correction before/after every trial; recalibration whenever drift occurred.
Piras et al. (2019)	Between groups comparison	Film-based	EyeLink II (SR Research) binocular video-based tracker; sampling rate 500 Hz; gaze resolution < 0.005°; noise < 0.01°; mounted on headband.	Fixation: gaze stable within 1° for ≥100 ms . Microsaccades: amplitude < 1°, following main-sequence velocity–amplitude relation; detected by unsupervised clustering ; must occur simultaneously in both eyes ≥3 samples (6 ms). Saccades: velocities 3°–100°/s; amplitude/peak velocity computed per IA. Calibration: 9-point grid; validation twice; drift correction after each trial if needed (p.4).	Recalibration every 10 trials; drift correction checked after every trial and applied when needed.
Rauniyar and Srivastava (2025)	Between-groups design	Film-based	Webcam-based eye tracker using GazeRecorder (online gaze tracking software); relies on standard laptop/iMac webcam	Calibration: implicit online calibration through webcam positioning; no formal multi-point calibration was described.	webcam-based system relies on initial face-tracking only.
Ripoll et al. (1995)	Between-groups comparison	Film-based	Nac Eye Mark Recorder V; accuracy ±1°;horizontal ±25° / vertical ±20° range;	Calibration: "The oculometer was calibrated before and after each sequence."	Accuracy was checked before and after each trial
Shirmehenji et al. (2019)	Causal-comparative design	Film-based	Pupil Labs eye tracker; 60 Hz sampling rate;recorded gaze points frame-by-frame.	Calibration:Five-point calibration before each clip;	Calibration before experiment; drift corrected visually throughout testing .
Singer et al. (1996)	Between-groups comparison	Film-based	Applied Sciences Laboratory Eye-Trac Model 210; infrared light + silicon phototransistors; horizontal & vertical gaze positions recorded; 32 cm monitor at 80 cm distance	Fixation definition: stationary ≥132 ms considered one fixationCalibration method: 9-point (3×3) electronic calibration chart; manual adjustment for offset/gain/linearity; recalibrated after each rest period	Recalibration conducted after each rest period (3 total)
Sáenz-Moncaleano et al. (2018)	Between groups comparison	In situation	SMI Eye Tracking Glasses 2 Wireless (ETG 2w); sampling rate 60 Hz; wireless recording; Smart Recorder worn at lower back; automatic event detection; scene camera used for ball-trajectory alignment.	Fixation definition: SMI ETG Event Detection algorithm; fixations = visual intake events after removing noise, saccades (>100°/s or >8°/s + skewness>5), blinks, undefined events <50 ms. Saccades: velocity-based classification per SMI algorithm. Calibration: standard 3-point calibration, repeated each block;	Calibration before task + repeated after each of the 4 blocks (3-point).
Takeuchi and Inomata (2009)	Between-groups design	In situation	Eye Mark Recorder Model EMR-8 (cap-mounted); precision < 0.1° horizontally & vertically; gaze cursor superimposed on video; recording at 50 Hz (video recorder).	Fixation: stationary within 1.5° tolerance ≥ 120 ms (≥6 video frames at 50 Hz). Calibration: cap-mounted EMR-8 calibrated before task;	Calibration before experiment; drift corrected visually throughout testing
Ward et al. (2002)	Mixed design	Film-based	ASL 5000SU eye tracker + Ascension Flock of Birds head tracker (6DFOB); 1° accuracy; allows 1.22m free movement	Fixation definition: fixation ≥100 ms within 1.5° movement toleranceCalibration: simple 9-point grid calibration before testing;	Calibration before each participant; periodic checks before familiarization & test films; no recalibration needed
Williams and Elliott (1999)	Between-groups comparison	Film-based	ASL 4000SU video-based eye-movement system;head-mounted gaze tracking via Iscan ASL 4000SU	Fixation definition: fixation = stationary eye for ≥2 frames(120 ms) Calibration: The system was calibrated using a nine-point reference	Major calibration performed once (after practice trials & before test trials); only occasional minor checks during experiment.
Witkowski et al. (2021)	Between-groups design	In situation	SMI Eye-Tracking Glasses (ETG 2 Natural Gaze); sampling rate 60 Hz; automatic parallax compensation; high-resolution scene camera (1280×960 px).	Calibration: standard 3-point calibration	Calibration conducted before the task using 3-point procedure; drift continuously compensated by the ETG system (no repeated recalibration reported)

### 3.2 Study characteristics

**Table 2**–**4** summarize the overall characteristics of the studies included in this review (n = 26). Overall, individual study sample sizes ranged from approximately 10 to 62 participants, with the smallest at 10 (Lin et al., 2021) and the largest at 62 (Hagemann et al., 2010) (**Table 2**). Regarding gender composition, 13 studies included both male and female athletes (Abernethy and Russell, 1987; Abernethy, 1990; Bandow and Witte, 2020, 2021; Chia et al., 2017; Guo et al., 2024; Hagemann et al., 2010; Murray and Hunfalvay, 2017; Nibbeling et al., 2012; Piras et al., 2014; Sáenz-Moncaleano et al., 2018; Singer et al., 1996; Witkowski et al., 2021); 6 studies included only males (Kato, 2020; Lin et al., 2021; Piras et al., 2019; Rauniyar and Srivastava, 2025; Takeuchi and Inomata, 2009; Williams and Elliott, 1999); 1 study included only females (Shirmehenji et al., 2015); and 6 did not explicitly report gender (Alder et al., 2014; Goh et al., 2018; Milazzo et al., 2016a; Ripoll et al., 1995; Ward et al., 2002; Goulet et al., 1989) (**Table 2**). In terms of competitive level grouping, all studies centered on “expert/elite/skilled—novice/less-skilled” comparisons, with a small number of incorporating multi-level or more complex designs, such as multi-level grouping or mixed/within-subjects designs (Hagemann et al., 2010; Kato, 2020). Participant samples consisted predominantly of young adult athletes, however, five studies included adolescent/underage samples (i.e., reported age information included <18 years) (Abernethy, 1990; Bandow and Witte, 2020, 2021; Goh et al., 2018; Witkowski et al., 2021) (**Table 2**).

In terms of sport distribution, the included studies spanned multiple disciplines, primarily focusing on tennis (n=5) (Lin et al., 2021; Murray and Hunfalvay, 2017; Sáenz-Moncaleano et al., 2018; Singer et al., 1996; Ward et al., 2002), badminton (n=4) (Abernethy and Russell, 1987; Alder et al., 2014; Chia et al., 2017; Shirmehenji et al., 2015), and karate (n=4) (Bandow and Witte, 2021, 2020; Milazzo et al., 2016a; Williams and Elliott, 1999), extending to fencing (n=2) (Guo et al., 2024; Witkowski et al., 2021), squash/orienteering/kendo/darts/judo/table tennis/mixed martial arts-boxing/savate/baseball (each n=1) (Abernethy, 1990; Rauniyar and Srivastava, 2025; Hagemann et al., 2010; Nibbeling et al., 2012; Piras et al., 2014; Piras et al., 2019; Takeuchi and Inomata, 2009; Ripoll et al., 1995; Singer et al., 1996) (**Table 3**). Regarding experimental tasks and settings 24 studies reported setting information). Of these, 14 employed video- or film-based paradigms (Abernethy and Russell, 1987; Alder et al., 2014; Bandow and Witte, 2020; Guo et al., 2024; Goulet et al., 1989; Lin et al., 2021; Nibbeling et al., 2012; Piras et al., 2014; Piras et al., 2019; Rauniyar and Srivastava, 2025; Ripoll et al., 1995; Sáenz-Moncaleano et al., 2018; Ward et al., 2002; Williams and Elliott, 1999), in situ/real-scene tasks in 11 studies (Bandow and Witte, 2021; Chia et al., 2017; Goh et al., 2018; Hagemann et al., 2010; Kato, 2020; Milazzo et al., 2016a; Murray and Hunfalvay, 2017; Takeuchi and Inomata, 2009; Singer et al., 1996; Nibbeling et al., 2012; Witkowski et al., 2021), and 1 study used both “video + in situ” settings (Abernethy, 1990) (**Table 4**).

Regarding outcomes and study themes 26 studies reported primary outcome themes, which focus on two main lines: (1)visual behavior (n=14) (Bandow and Witte, 2021, 2020; Chia et al., 2017; Lin et al., 2021; Murray and Hunfalvay, 2017; Nibbeling et al., 2012; Piras et al., 2014; Piras et al., 2019; Rauniyar and Srivastava, 2025; Ripoll et al., 1995; Sáenz-Moncaleano et al., 2018; Takeuchi and Inomata, 2009; Williams and Elliott, 1999; Goh et al., 2018); and (2) decision-making/prediction performance (n=12) (Abernethy and Russell, 1987; Abernethy, 1990; Alder et al., 2014; Guo et al., 2024; Hagemann et al., 2010; Singer et al., 1996; Ward et al., 2002; Shirmehenji et al., 2015; Nibbeling et al., 2012; Piras et al., 2014; Sáenz-Moncaleano et al., 2018; Goulet et al., 1989). At the secondary outcome/specific eye movement indicator level, studies most commonly described and compared fixation count, fixation duration, and fixation location, extending to number of fixation locations (n=8) (Bandow and Witte, 2020; Chia et al., 2017; Kato, 2020; Milazzo et al., 2016a; Murray and Hunfalvay, 2017; Nibbeling et al., 2012; Singer et al., 1996; Williams and Elliott, 1999), fixation location distribution/key AOIs (n=5) (Alder et al., 2014; Bandow and Witte, 2020; Chia et al., 2017; Murray and Hunfalvay, 2017; Rauniyar and Srivastava, 2025), visual pivot/visual anchor (n=1) (Kato, 2020), quiet eye duration (n=3) (Chia et al., 2017; Goh et al., 2018; Sáenz-Moncaleano et al., 2018), and a few incorporating verbal reports as process measures (Milazzo et al., 2016a) (**Table 3**).

### 3.3 Risk of bias in individual studies

This review used Review Manager (RevMan) software to organize and visualize the risk of bias for the included studies, employing the RoBANS (Risk of Bias Assessment tool for Non-randomized Studies) as the assessment framework (**Figure 1**). RoBANS comprises six domains: participant selection, confounding variables, consistency of exposure/measurement, blinding of outcome assessment, incomplete outcome data, and selective outcome reporting. Each domain was reated as “low risk,” “high risk,” or “unclear” (Kim et al., 2013). Risk of bias assessments were completed independently by two reviewers, and any disagreements were resolved through discussion.

Based on the RevMan risk of bias summary results: In the domain of participant selection, the majority of studies were rated as low risk (20/26), with the remaining studies rates as unclear (6/26). The domain of confounding variables showed relatively greater concern, with only 4/26 studies rated as low risk, 5/26 as high risk, and most judged unclear (17/26) due to insufficient reporting of covariate control or familiarization procedures. In the domain of exposure/measurement consistency, most studies reported relatively clear eye-tracking procedures and consistent task conditions, resulting in predominantly low risk ratings (24/26), with only 2/26 unclear. In terms of blinding of outcome assessment, 17/26 studies were rates as low risk, whereas 9/26 remained unclear beacuse of inadequate description of data cleaning, AOI labeling, or interrater reliability. Regarding incomplete outcome data, 17/26 were rated as low risk, while 9/26 were judged as unclear owing to insufficient information on missing/excluded data or handling of tracking quality issues. In the domain of selective outcome reporting, 17/26 were rated as low risk, however, 9/26 were difficult to judge due to the absence of comparable preregistration/protocol or inadequate reporting details.

Overall, the studies included in this review performed relatively well in the domains of “participant selection” and “exposure/measurement consistency.” However, inadequate control and transparent reporting in the “confounding variables” domain represent the primary methodological limitation, which must be fully considered when interpreting conclusions regarding expert–novice differences (Kim et al., 2013).

### 3.4 Chronological age

**Figure 3:**
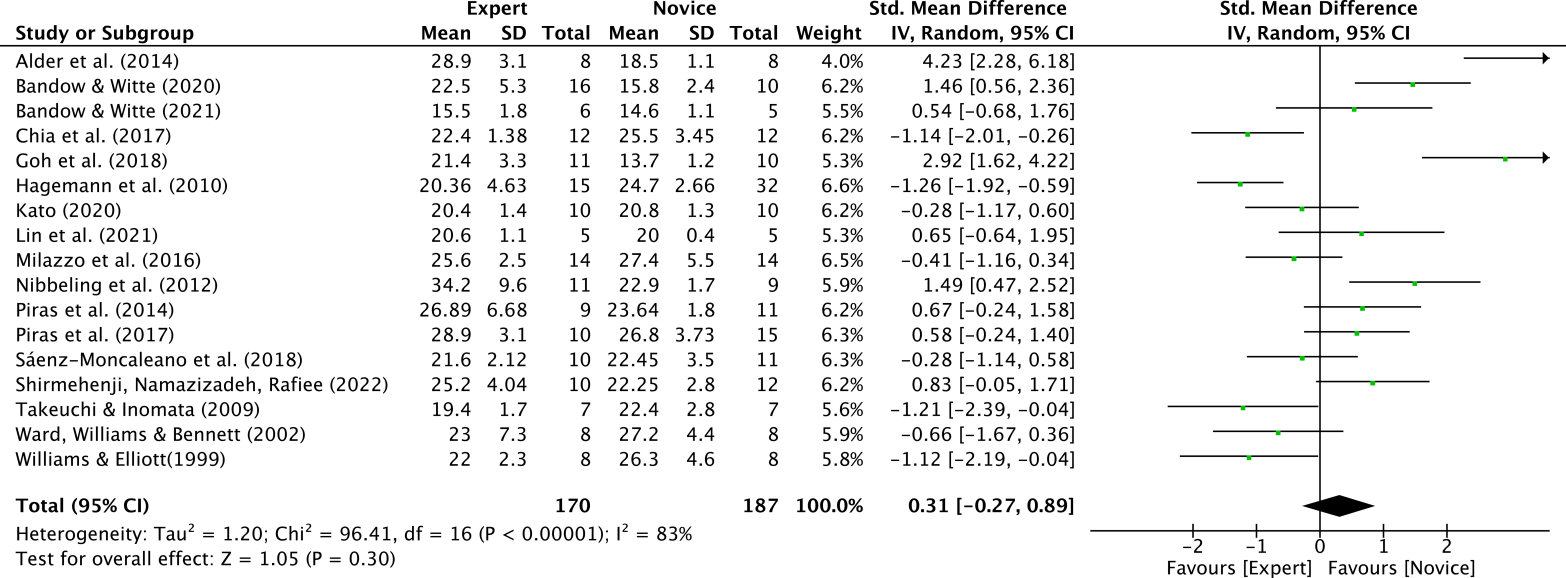
Chronological age: similar chronological age was noted for expert athletes compared to novice athletes. Green squares: individual studies. Its size represents their relative weights. Black rhomboid: summary value.

A total of 17 studies reported age data suitable for meta-analysis, contributing 17 expert groups and 17 novice groups (expert n = 170; novice n = 187; total N = 357; Alder et al., 2014; Bandow and Witte, 2020, 2021; Chia et al., 2017; Goh et al., 2018; Hagemann et al., 2010; Kato, 2020; Lin et al., 2021; Milazzo et al., 2016a; Nibbeling et al., 2012; Piras et al., 2014; Piras et al., 2019; Sáenz-Moncaleano et al., 2018; Shirmehenji et al., 2019; Takeuchi and Inomata, 2009; Ward et al., 2002; Williams and Elliott, 1999). Results from Review Manager (RevMan) indicated a moderate effect size. Expert athletes were slightly older than novices, but the overall difference did not reach statistical significance (SMD = 0.31; 95% CI = −0.27 to 0.89; Z = 1.05, p = 0.30) (**Figure 3**). τ² = 1.20; χ² = 96.41, df = 16, p < 0.00001; I² = 83%; Begg’s test = 0.0638.

**Figure 4:**
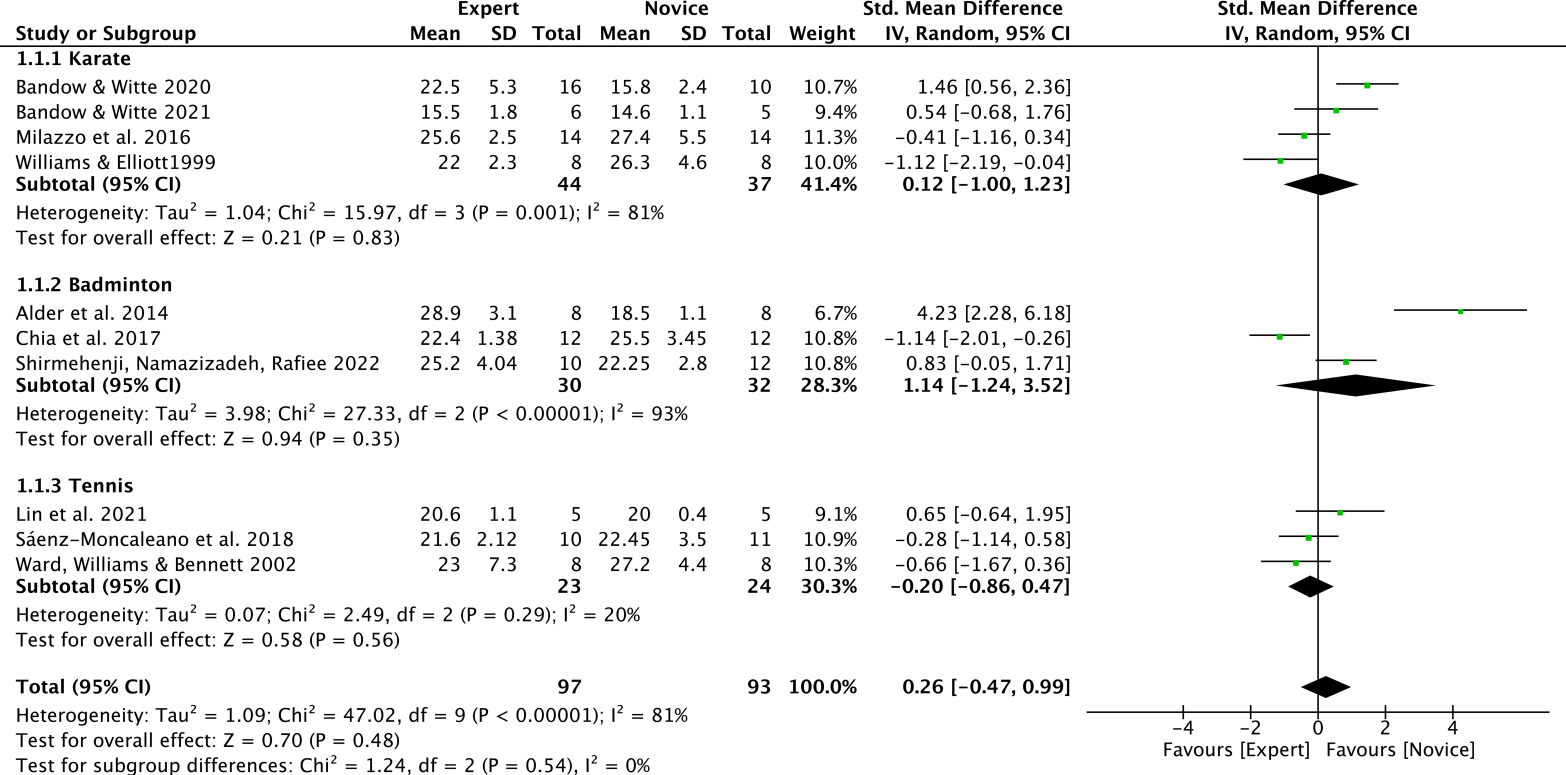
Chronological age moderated by type of sport: no significant moderator effect was noted for the type of sport (p = 0.69 between groups).

**Figure 5:**
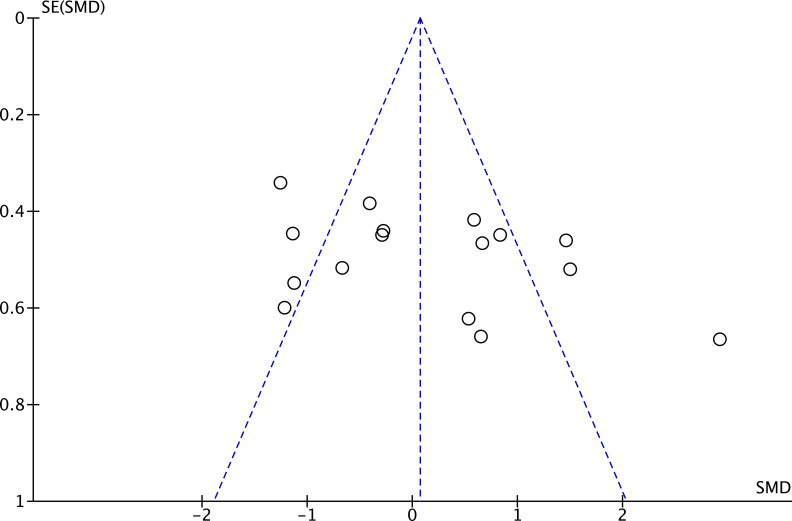
Funnel plot for chronological age: The individual study points are symmetrically distributed around the pooled effect size (dashed line), showing no evident signs of publication bias.

A subgroup analysis by sport type was conducted, including only sports with at least three available studies (karate: 4 studies; badminton: 3 studies; tennis: 3 studies; total experts n = 97, novices n = 93). Sport type did not significantly moderate the effect (between-group χ² = 1.24, df = 2, p = 0.54; I² = 0%), and pooled effects within each subgroup were non-significant: karate SMD = 0.12 (95% CI = −1.00 to 1.23), badminton SMD = 1.14 (95% CI = −1.24 to 3.52), tennis SMD = −0.20 (95% CI = −0.86 to 0.47) (**Figure 4**).Other sports were not included in the moderator analysis, as less than three studies were available. Additionally, the funnel plot showed a relatively concentrated distribution, with no evident asymmetry overall (**Figure 5**).

### 3.5 Years of experience

**Figure 6:**
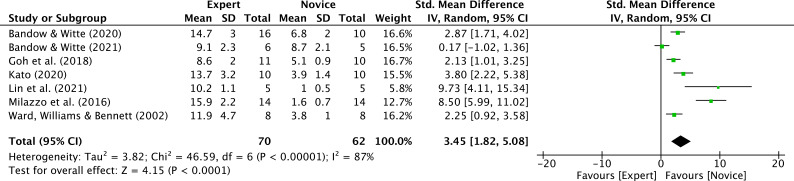
Years of experience: greater years of experience were noted for expert athletes compared to novice athletes. Green squares: individual studies. Its size represents their relative weights. Black rhomboid: summary value.

Six studies reported data on years of practice, involving 7 expert groups and 7 novice groups (pooled n = 62) (Bandow and Witte, 2020, 2021; Goh et al., 2018; Kato, 2020; Lin et al., 2021; Milazzo et al., 2016a; Ward et al., 2002). The results indicated an extremely large effect size. Compared with novice athletes, expert athletes had significantly longer years of practice (SMD = 3.45; 95% CI = 1.82 to 5.08; Z = 4.15, p < 0.0001) (**Figure 6**). τ² = 3.82; χ² = 46.59, df = 6, p < 0.00001; I² = 87%. Moderator analyses as per athletes’ type of sport were precluded, as less than three studies were available.

### 3.6 Number of fixations

**Figure 7:**
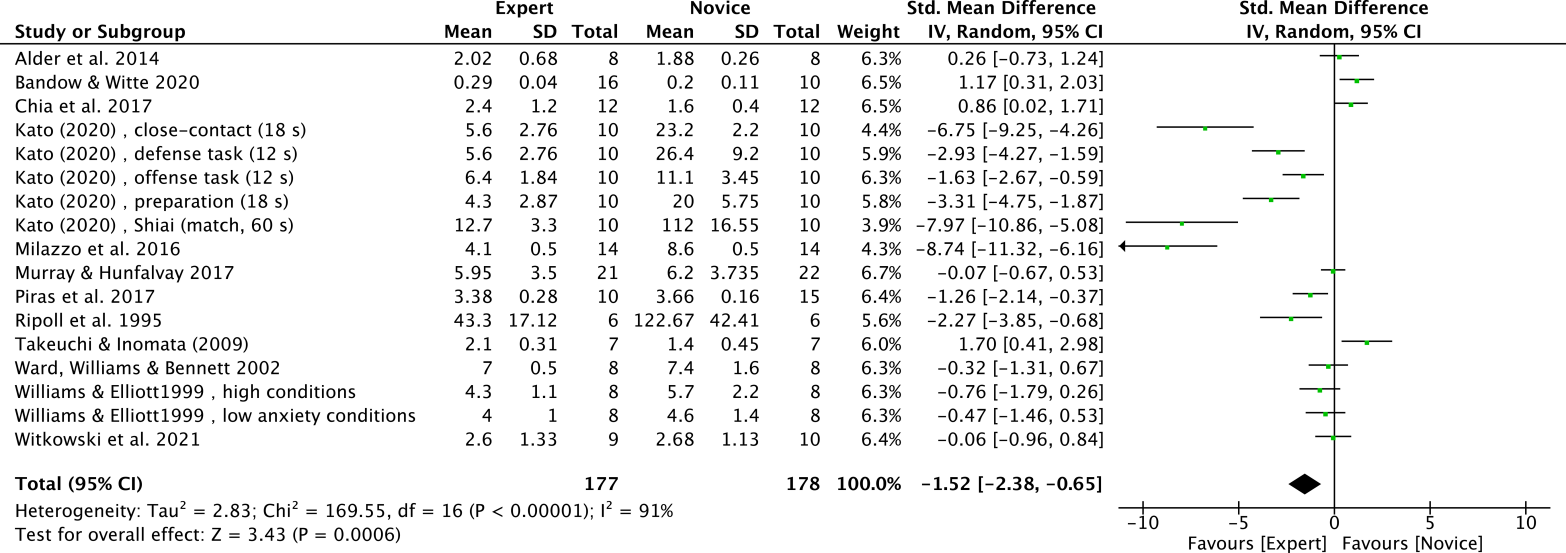
Number of fixations: Fewer number of fixations were noted for expert athletes compared to novice athletes. Green squares: Individual studies. Its size represents their relative weights. Black rhomboid: summary value.

**Figure 8:**
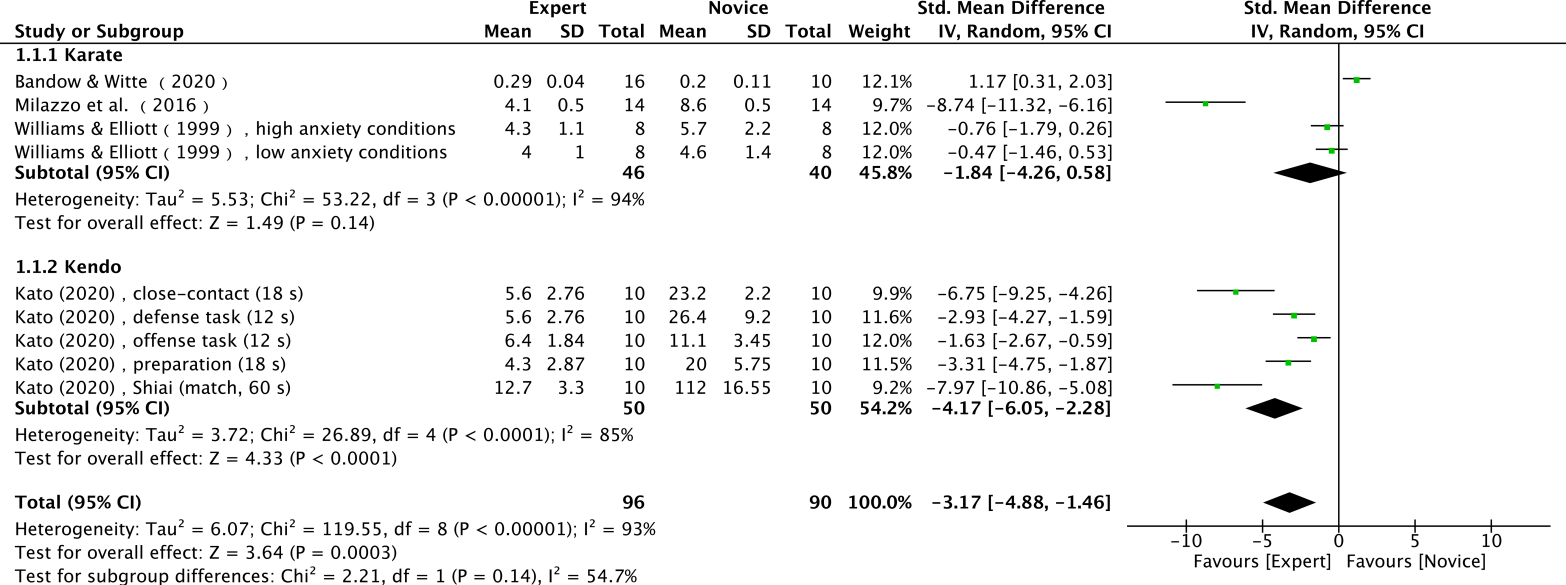
Number of fixations moderated by type of sport: no significant moderator effect was noted for type of sport (p = 0.14 between groups).

**Figure 9:**
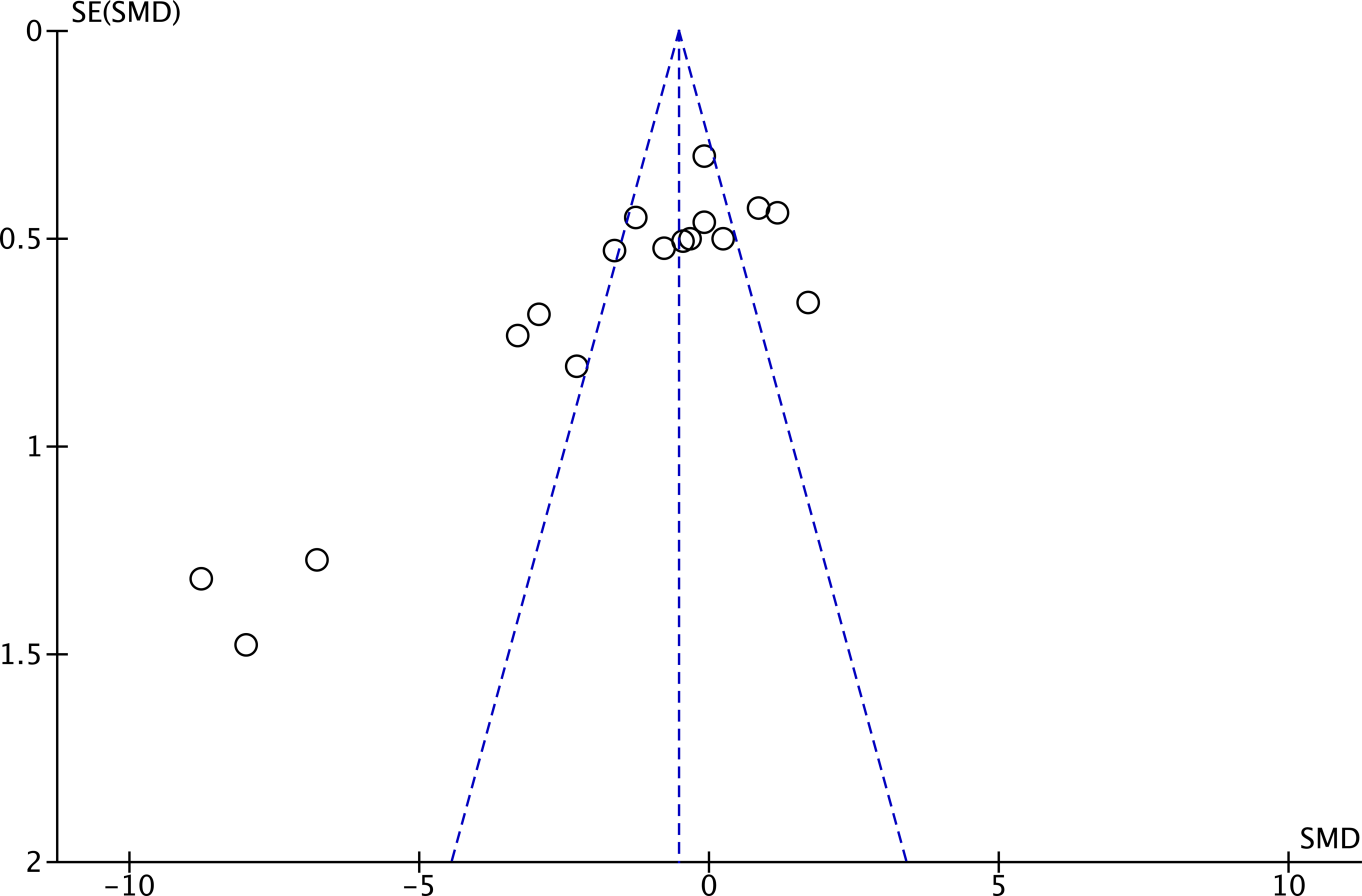
Funnel plot for number of fixations: Most of the study points are distributed symmetrically around the pooled effect size (dashed line), with no evident signs of publication bias.

Twelve studies (17 effect sizes) provided number of fixations data, comprising 17 expert groups and 17 novice groups (combined total of 355 participants) (Alder et al., 2014; Bandow and Witte, 2020; Chia et al., 2017; Kato, 2020; Milazzo et al., 2016a; Murray and Hunfalvay, 2017; Piras et al., 2019; Ripoll et al., 1995; Takeuchi and Inomata, 2009; Ward et al., 2002; Williams and Elliott, 1999; Witkowski et al., 2021). The results showed a very large effect size, with expert athletes showing significantly fewer number of fixations compared to novices (SMD = −1.52; 95% CI = −2.38 to −0.65; Z = 3.43, p = 0.0006) (**Figure 7**). τ² = 2.83; χ² = 169.55, df = 16, p < 0.00001; I² = 91%. Funnel plot visual inspection showed that most data points were clustered in the center, suggesting stable effect estimates from these studies (**Figure 8**). Further subgroup analysis by sport type included only those with at least three available effect sizes. The karate subgroup showed no significant difference (SMD = −1.84; 95% CI = −4.26 to 0.58; p = 0.14), while the kendo subgroup showed significantly fewer number of fixations for experts (SMD = −4.17; 95% CI = −6.05 to −2.28; p < 0.0001). However, between-group analysis indicated that the moderating effect of sport type was not significant (χ² = 2.21, df = 1, p = 0.14) (**Figure 9**). Other sports were not included in the moderator analysis, as less than three studies were available.

### 3.7 Fixation duration

**Figure 10:**
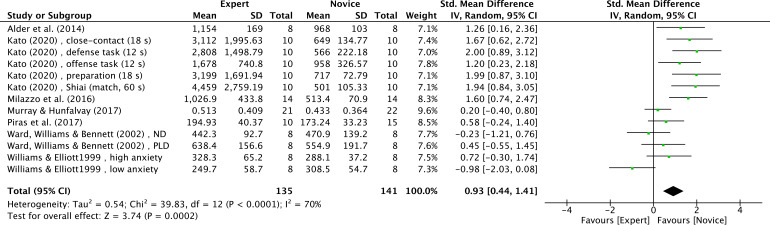
Fixation duration: longer fixation duration was noted for expert athletes compared to novice athletes. Green squares: individual studies. Its size represents their relative weights. Black rhomboid: summary value.

Seven studies (13 effect sizes) (Alder et al., 2014; Kato, 2020; Milazzo et al., 2016a; Murray and Hunfalvay, 2017; Piras et al., 2019; Ward et al., 2002; Williams and Elliott, 1999) provided fixation duration data for expert and novice groups, with a total sample size of 276 participants. The results indicated a very large effect size, with expert athletes showing significantly longer fixation durations compared to novices (SMD = 0.93; 95% CI = 0.44 to 1.41; Z = 3.74, p = 0.0002) (**Figure 10**). τ² = 0.54, χ² = 39.83, df = 12, p < 0.0001, I² = 70%. Moderator analyses as per athletes’ type of sport were precluded, as less than three studies were available.

### 3.8 Number of fixation locations

**Figure 11:**
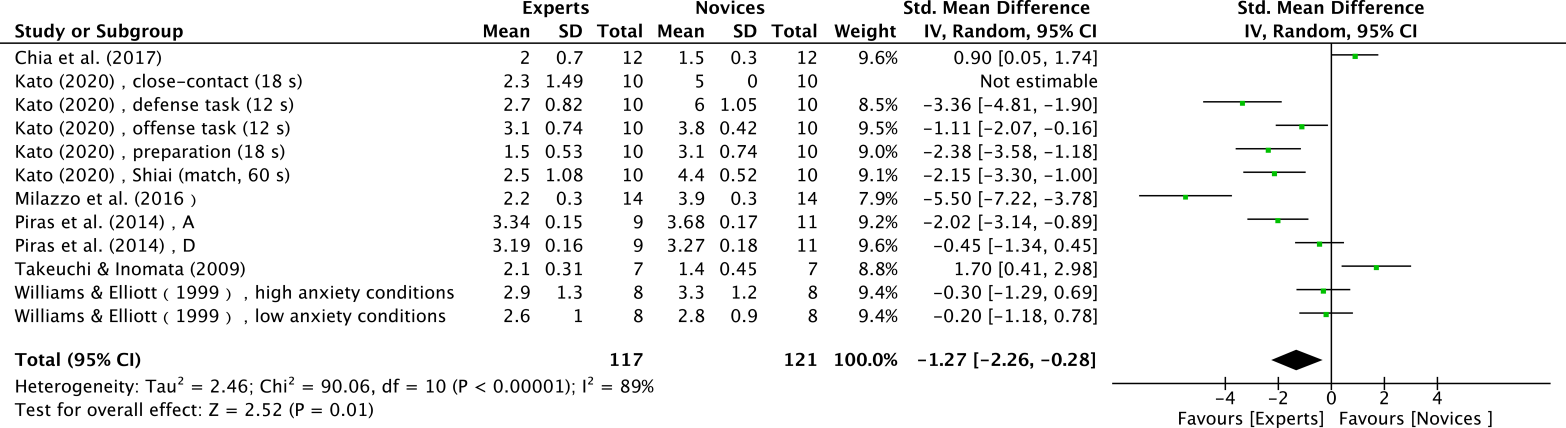
Number of fixation locations: lower number of fixation locations was noted for expert athletes compared to novice athletes. Green squares: individual studies; Its size represents their relative weights. Black rhomboid: summary value.

Six studies contributed data on the number of gaze locations(11 effect sizes), comprising expert and novice groups with a total sample of 218 individuals (Chia et al., 2017; Kato, 2020; Milazzo et al., 2016a; Piras et al., 2014; Takeuchi and Inomata, 2009; Williams and Elliott, 1999). The results showed a very large effect size, with experts fixating on fewer locations than novices (SMD = -1.27; 95% CI = -2.26 to -0.28; Z = 2.52, p = 0.01) (**Figure 11**). τ² = 2.46, χ² = 90.06, df = 10, p < 0.0001, I² = 89%. Moderator analyses as per athletes’ sport type were not conducted as less than three studies were available.

### 3.9 Fixation locations

Five studies investigated fixation locations. Four studies reported significant differences in fixation locations between experts and novices (Alder et al., 2014; Chia et al., 2017; Murray and Hunfalvay, 2017; Ripoll et al., 1995). One study found no significant group difference (Bandow and Witte, 2020).

### 3.10 Quiet eye duration

Three studies examied quiet eye duration, involving three expert groups and three novice groups (combined n = 61). Two studies found that experts had longer quiet eye durations than in novices (Goh et al., 2018; Goulet et al., 1989). The other study showed the opposite result (Sáenz-Moncaleano et al., 2018).

### 3.11 Other outcomes

This review initially planned to include several secondary outcome measures related to visual behavior, including but not limited to: number of fixations, fixation duration, fixation locations and their distribution, search rate, prediction and decision accuracy, reaction time, quiet eye characteristics, scan paths, fixation transitions, as well as saccade and microsaccade features. However, during the data extraction process, it was found that although some studies reported outcomes such as fixation duration at individual locations, fixation order/sequence, visual field coverage (including central and peripheral vision), and the amplitude, duration, peak velocity, and acceleration of microsaccades or saccades, these outcomes could not be quantitatively combined for analysis. This was primarily due to inconsistent reporting formats, incomplete statistical information (such as the lack of means, standard deviations, or sample sizes), or the results being presented only in qualitative descriptions or illustrations. Consequently, these outcome measures were not included in the meta-analysis as initially planned and were not further explored in the subsequent synthesis and discussion of results.

## 4 Discussion

This systematic review and meta-analysis examined expert–novice differences in visual search behaviour. As expected, the results showed significant differences between experts and novices on most visual search indicators. These differences reflect the more efficient visual search strategies employed by expert athletes (Kato, 2020; Rayner, 1998), an advantage closely linked to their superior underlying cognitive processing. Event-related potential (ERP) research further elucidates this intrinsic mechanism, demonstrating that experts generate significantly larger P3 amplitudes during task execution( (Aly et al., 2019, 2024)). This neurophysiological marker suggests a more effective allocation of attentional resources and accelerated identification and integration of task-relevant information (Aly et al., 2019, 2024). Ultimately, such neural-level processing efficiency serves as the core driver enabling experts to optimize their visual search behaviors in demanding sporting environments.

### 4.1 Chronological age and years of practice

This study conducted a meta-analysis on chronological age (n=17) and years of practice (n=6). The results showed that chronological age had a moderate effect (though not significant, P = 0.3), while years of practice showed a very large effect (Z = 4.15, p < 0.0001). Compared to novices, experts had greater chronological age and more years of practice. Silva et al. (2022) suggested that this may be due to the absence of a clear definition of expertise. Swann et al. (2015) highlighted that at least eight different criteria have been used to define expert athletes. Among these, the most commonly applied criterion to distinguish expert from non-expert athletes is participation in international and/or national-level competitions, followed by the athlete’s experience, with professional characteristics ranked third.

### 4.2 Number of fixations

The number of fixations refers to the count of fixations identified within a given time period (Mahanama et al., 2022). Fixations can be counted across the entire stimulus range or within a single area of interest (AOI) (Holmqvist et al., 2011). In this study, a meta-analysis of 12 studies showed significant differences in the number of fixations (Z = 3.43, p = 0.0006, **Figure 7**). with experts making fewer fixations than novices.

These findings may be explained by two complementary mechanisms. First, extensive practice allows experts to accumulate sufficient task-related information in their long-term working memory (LTM-WM). When performing a specific task, experts can rapidly and efficiently retrieve this internalized information, without needing to make more fixations to obtain relevant details (Rayner, 1998), which is highly consistent with the long-term working memory theory proposed by Ericsson and Kintsch (1995). Second, Milazzo et al. (2016b) found that, unlike novices, experts do not rely on central vision (fovea) to scan peripheral areas but instead directly use peripheral vision to extract information. This result is consistent with findings by Piras et al. (2014), which found that, although expert judoka made fewer fixations compared to novices, they extracted more task-relevant information during each fixation.

However, Chia et al. (2017) reported that expert badminton players made more fixations than novices during the serve. One possible explanation is that the serve is a self-paced task in badminton,providing athletes with sufficient time to prepare and allocate visual attention. Additionally, technically skilled players may already be accustomed to the importance of the serve and, therefore, act more cautiously, which might lead to more fixations by experts.

### 4.3 Mean fixation duration

Fixation duration refers to the length of time the eyes remain stationary at a particular location (Salvucci and Goldberg, 2000). Researchers typically determine the focus of attention by calculating the average fixation duration across different areas of interest (AOIs) (Goldberg and Kotval, 1999).

In this study, a meta-analysis of 7 studies showed a significant difference in fixation duration between experts and novices (Z = 3.74, p = 0.0002) (**Figure 10**), with experts exhibiting longer fixation durations. Several factors may account for these findings. First, the expert advantage is theoretically rooted in more efficient underlying cognitive processing. Aly et al. (2024) demonstrated that athletes exhibit significantly larger P3 amplitudes compared to amateurs when processing task-relevant information [F(2, 90) = 15.07, p < 0.01, η2 ≥ 0.25]. As a core neurophysiological marker of attentional allocation and stimulus evaluation speed, the enhanced P3 component indicates that experts possess superior attentional engagement and more efficient resource dispatching capabilities (Aly et al., 2019, 2024). This neural efficiency may help explain expert–novice differences in the timing of information extraction reflected in fixation behaviour; specifically, larger P3 amplitudes may indicate faster and higher-quality integration of task-relevant information within a given fixation.

Second, this heightened neural engagement likely reflects deeper cognitive processing by experts (Rayner, 1978; Salthouse and Ellis, 1980). This perspective is supported by Meghanathan et al. (2015), who found that fixation duration increases under higher cognitive load. In the sporting-specific context, Alder et al. (2014) suggested that longer fixation durations grant badminton experts more time to extract critical information from their opponent’s body kinematics. Consequently, these findings align with the established ‘expert performance’ profile noted by Williams and Elliott (1999), where experts tend to employ a more efficient visual strategy characterized by fewer fixations of longer durations than novices.

However, visual search patterns often vary depending on the sports, task, and potential context (Afonso et al., 2014). In the meta-analysis by Silva et al. (2022), most studies reported no significant difference in fixation duration between experts and novices, which may be due to differences across sports. Vater et al. (2020) found that,Vater et al. (2020) showed that in team-sport settings, experts tend to produce a greater number of short-duration fixations than novices.

### 4.4 Number of fixation locations

In this study, a meta-analysis of 6 studies showed a significant difference (Z = 2.52, p = 0.01) (**Figure 11**), with experts having fewer fixation locations compared to novices.

Two possible reasons for this may be proposed. First, extensive practice enables experts to develop a large repository of task-relevant information, allowing them to ignore irrelevant details and effectively focus on key elements. This ability enables experts to obtain more information with fewer fixation locations (Pieters et al., 1997). Second, the concept of “gaze anchor” proposed by Vater et al. (2020) can explain this phenomenon. By fixing their gaze on specific locations and using peripheral vision to process information, experts effectively eliminate the time and effort costs associated with saccadic eye movements. In contrast, novices, whose visual search strategies are characterized by heightened alertness, tend to sweep their gaze more broadly and frequently in an attempt to quickly detect potentially threatening cues (Eysenck, 1992), leading to a significantly higher number of fixation locations during their visual search. This result is supported by Milazzo et al. (2016b), who found that expert karate athletes change their visual search behavior by concentrating their attention on fewer locations with longer durations.

However, Chia et al. (2017) argued that, experts make more fixations than novices. This visual search pattern of experts may not be passive information gathering but rather a deliberate strategy. Specifically, during the serve, experts switches fixations between multiple locations to reduce the available predictive cues for the opponent, thereby decreasing the opponent’s ability to predict the serve’s direction and/or landing spot (Jackson et al., 2006).

### 4.5 Quiet eye duration

The quiet eye (QE) is operationally defined as the final stable fixation on a specific target lasting at least four frames, prior to the initiation of the forearm movement and the ball racket contact (Vickers, 1996).

Among the included studies, three studies examnied quiet eye duration (Chia et al., 2017; Goh et al., 2018; Sáenz-Moncaleano et al., 2018). Two studies (Goh et al., 2018; Sáenz-Moncaleano et al., 2018) reported significant differences between experts and novices (P = 0.01; P = 0.037), with experts showing longer quiet eye durations. Longer quiet eye durations may indicate that athletes have more time to “program” and make subtle adjustments to their responses (Vickers, 2009), which is a typical characteristic of expert performance. Additionally, longer quiet eye durations are often associated with an extended key response preparation period, which may help tennis players make more appropriate responses and further fine-tune parameters such as power, timing, and ball direction during the motor programming process (Moore et al., 2012).

### 4.6 Fixation locations

Five studies reported differences in fixation locations between experts and novices (Alder et al., 2014; Bandow and Witte, 2020; Chia et al., 2017; Murray and Hunfalvay, 2017; Ripoll et al., 1995). More studies (n=4) indicated that, compared to novices, experts mostly focus on key body parts, such as the head and chest, and pay less attention to distal body parts.

One possible explanation is that experts are better able to fixate their gaze between a few cues and visual information, using and shifting covert attention rather than overt attention, in order to gather more visual information (Vater et al., 2016; Vater et al., 2017b). Additionally, this may result from the interaction between central vision and peripheral vision. Hausegger et al. (2019) proposed a context-dependent gaze anchoring strategy, where experts fix their gaze on a relatively “central/information-dense” area, such as the chest or abdomen, as a gaze anchor, while simultaneously using peripheral vision to monitor changes in nearby body parts like the arms, head, and legs. Furthermore, this gaze anchoring may change over time or with changing circumstances. This result supports previous related studies (Williams and Elliott, 1999; Milazzo et al., 2016b; Mori et al., 2022; Rosalie and Muller, 2013).

However, Alder et al. (2014) found that, compared to novices, expert badminton players are more likely to fixate on the racket. This may be because key cues used to distinguish the type of stroke primarily come from more distal kinematic areas, such as the racket and wrist.

In addition, a subgroup analysis was conducted to examine whether sport type moderated the effect of expertise on visual search behaviour. The results indicated that sport type (i.e., karate, badminton, and tennis) did not significantly moderate the impact of expertise on visual search behavior (df = 2, p = 0.54;I^2^ = 0%). Furthermore, the pooled effects within each sport subgroup were also non-significant. One possible explanation is that, across both open-skill (e.g., tennis, karate) and closed-skill sports, athletic experience enhances attentional processing efficiency by increasing P300 amplitudes and shortening latencies (Aly et al., 2019, 2024). This is consistent with prior research suggesting that both open- and closed-skill training exert comparable positive effects on visuospatial attention and processing speed (Chueh et al., 2017; Giglia et al., 2011; Guo et al., 2016).

### 4.7 Generalizability and transfer of perceptual-cognitive expertise

Furthermore, the findings of this study offer significant implications for general skill acquisition. Evidence suggests that the attentional advantages associated with motor expertise may not be task-specific but could potentially transfer to non-sporting contexts, such as general visual or auditory attention tasks (Aly et al., 2019, 2024). This potential for transfer supports the broader applicability of perceptual-cognitive expertise. It indicates that long-term motor training not only optimizes sport-specific performance but may also enhance general information-processing capabilities in daily life and complex environments by modulating underlying neurophysiological mechanisms, such as more efficient P300-related attentional resource allocation.

### 4.8 Limitations

Several limitations of the present study should be acknowledged when interpreting these findings.

First, the definition and classification of “expert” and “novice” athletes varied considerably across the included studies. Expertise was operationalized using a range of criteria, including participation in international/national competitions, years of deliberate practice, competitive ranking, or subjective coach evaluations (Swann et al., 2015; McKay et al., 202x2). This methodological heterogeneity may introduce confounding and reduce the comparability of effect sizes across studies, therebt potentially inflating or masking true expert-novice differences.

Second, the number of studies available for serveral outcomes was relatively small (e.g., 6 studies for years of practice, 7 for fixation duration, and 3 for quiet eye duration). This limited sample size prevented comprehensive subgroup and moderator analyses (e.g., by sport type, gender, age group, or experimental setting) and reduced statistical power for detecting potential moderating effects.

Third, risk-of-bias assessment using RoBANS revealed moderate methodological concerns, particularly in the control of confounding variables. Many studies did not adequately match or statistically control for age, gender, anxiety levels, or other potential confounders, and detailed reporting of eye-tracker calibration procedures, data cleaning, and AOI definition was often incomplete. These limitations may have introduced bias into the estimated expert–novice differences.

Fourth, most studies relied on laboratory-based video occlusion tasks or highly controlled *in-situ* settings, which limits the ecological validity. The high-pressure, dynamic, and physically demanding nature of real competition—where body movement, fatigue, and opponent deception are prominent—was underrepresented, potentially limiting the generalizability of findings to actual competitive performance.

These limitations highlight the need for future research employing standardized expertise criteria, larger sample sizes, more consistent reporting of eye-tracking metrics, and higher ecological validity paradigms to further validate and extend the current conclusions.

## 5 Conclusion

In conclusion, this systematic review and meta-analysis provides evidence that expert athletes in individual sports exhibit distinct and more efficient visual search behaviours compared to novices. Experts demonstrate significantly longer years of practice, fewer fixations, longer fixation durations, and fewer fixation locations, reflecting optimized perceptual-cognitive processing that allows for rapid extraction of task-relevant cues while minimizing unnecessary eye movements. These patterns are consistent with theories of long-term working memory and gaze anchoring, enabling experts to leverage peripheral vision for broader environmental monitoring without sacrificing focus on critical areas like the head and torso. Although subgroup analyses by sport type did not indicate significant moderation, the consistency across diverse individual disciplines—such as tennis, badminton, and karate—underscores the generalizability of these advantages.

The findings have practical implications for sports training, suggesting that interventions targeting visual search strategies (e.g., quiet eye training or occlusion-based paradigms) may accelerate skill acquisition and enhance performance in novices. However, several limitations warrant consideration: the heterogeneity in how “expertise” was defined (e.g., varying criteria like competition level or practice hours) may have confounded between-study comparisons, and the small number of studies for certain outcomes (e.g., quiet eye) limited comprehensive meta-analyses. Additionally, biases in confounding variables and incomplete methodological reporting in included studies warrant caution in interpretation.

Future research should adopt standardize expertise classifications, incorporate advanced eye-tracking metrics (e.g., microsaccades), and examine key moderating factors such as gender, age, and anxiety in larger, longitudinal designs. By bridging the gap between team and individual sports literature, this study lays a foundation for evidence-based perceptual training, ultimately contributing to improved athletic decision-making and performance.

## Data Availability

The original contributions presented in the study are included in the article/supplementary material. Further inquiries can be directed to the corresponding author.
